# Targeting triple-negative breast cancer using cord-blood CD34⁺ HSPC-derived mesothelin-specific CAR-NKT cells with potent antitumor activity

**DOI:** 10.1186/s13045-025-01736-9

**Published:** 2025-10-13

**Authors:** Yan-Ruide Li, Xinyuan Shen, Yichen Zhu, Zhe Li, Ryan Hon, Yanxin Tian, Jie Huang, Annabel S. Zhao, Nathan Y. Ma, Catherine Zhang, David Lin, Karine Sargsyan, Yuan Yuan, Lili Yang

**Affiliations:** 1 Department of Microbiology, Immunology & Molecular Genetics, University of California, Los Angeles, Los Angeles, CA 90095 USA; 2 Department of Bioengineering, University of California, Los Angeles, Los Angeles, CA 90095 USA; 3 Division of Medical Oncology, Cedars-Sinai Medical Center, Los Angeles, CA 90048 USA; 4 OncoBiobank, Cedars-Sinai Medical Center, Los Angeles, CA 90048 USA; 5 Eli and Edythe Broad Center of Regenerative Medicine and Stem Cell Research, University of California, Los Angeles, Los Angeles, CA 90095 USA; 6 Jonsson Comprehensive Cancer Center, David Geffen School of Medicine, University of California, Los Angeles, Los Angeles, CA 90095 USA; 7 Molecular Biology Institute, University of California, Los Angeles, Los Angeles, CA 90095 USA; 8 Parker Institute for Cancer Immunotherapy, University of California, Los Angeles, Los Angeles, CA 90095 USA; 9 Goodman-Luskin Microbiome Center, University of California, Los Angeles, Los Angeles, CA 90095 USA

**Keywords:** Triple-negative breast cancer (TNBC), Allogeneic CAR-NKT cells, Mesothelin-targeting CAR (MCAR), Allogeneic cell therapy, Off-the-shelf, Potent antitumor activity, Tumor microenvironment (TME), Tumor-associated macrophage (TAM), Multiple tumor targeting mechanism, Orthotopic model, Metastatic model, CRISPR-Cas9 gene editing, Allorejection, HLA ablation

## Abstract

**Background:**

Triple-negative breast cancer (TNBC) is an aggressive subtype of breast cancer characterized by the lack of ER, PR, and HER2 expression. Its aggressive behavior, high degree of tumor heterogeneity, and immunosuppressive tumor microenvironment (TME) are associated with poor clinical outcomes, rapid disease progression, and limited therapeutic options. Although chimeric antigen receptor (CAR)-engineered T cell therapy has shown certain promise, its applicability in TNBC is hindered by antigen escape, TME-mediated suppression, and the logistical constraints of autologous cell production.

**Methods:**

In this study, we employed hematopoietic stem and progenitor cell (HSPC) gene engineering and a feeder-free HSPC differentiation culture to generate allogeneic IL-15-enhanced, mesothelin-specific CAR-engineered invariant natural killer T (^Allo15^MCAR-NKT) cells.

**Results:**

These cells demonstrated robust and multifaceted antitumor activity against TNBC, mediated by CAR- and NK receptor-dependent cytotoxicity, as well as selective targeting of CD1d^+^ TME immunosuppressive cells through their TCR. In both orthotopic and metastatic TNBC xenograft models, ^Allo15^MCAR-NKT cells demonstrated potent antitumor activity, associated with robust effector and cytotoxic phenotypes, low exhaustion, and a favorable safety profile without inducing graft-versus-host disease.

**Conclusions:**

Together, these results support ^Allo15^MCAR-NKT cells as a next-generation, off-the-shelf immunotherapy with strong therapeutic potential for TNBC, particularly in the context of metastasis, immune evasion, and treatment resistance.

**Supplementary Information:**

The online version contains supplementary material available at 10.1186/s13045-025-01736-9.

## Background

Breast cancer is the most diagnosed cancer in women and remains the leading cause of death from malignant tumors among women; in the US, about 1 in 8 women will develop invasive breast cancer over the course of their lifetime [1]. Triple-negative breast cancer (TNBC) is a highly aggressive subtype of breast cancer defined by the absence of estrogen receptor (ER), progesterone receptor (PR), and HER2 overexpression [2]. Representing approximately 15–20% of all breast cancers, TNBC is associated with poor clinical outcomes, rapid progression, and limited treatment options in the advanced setting [1, 3, 4]. While chemotherapy remains the backbone of therapy for advanced TNBC, its efficacy is hampered by tumor heterogeneity and resistance mechanisms, yielding a median overall survival (OS) of only 18–24 months in the metastatic setting [1]. Although recent advances have introduced immune checkpoint inhibitors (ICIs) and antibody-drug conjugates (ADCs) to the TNBC treatment landscape, the overall benefit remains modest, and novel therapeutic strategies are urgently needed [1].

Chimeric antigen receptor (CAR)-engineered T (CAR-T) cell therapy has emerged as a powerful tool for treating hematologic malignancies and is now being actively investigated in solid tumors such as TNBC [5–8]. Several tumor-associated antigens, including mesothelin (MSLN; NCT02414269, NCT02580747, and NCT02792114), trophoblast cell-surface antigen 2 (TROP2; NCT06066424), Mucin 1 (MUC1; NCT02587689 and NCT04020575), epidermal growth factor receptor (EGFR; NCT05341492), and Nectin-4 (NCT06724835), have been identified as viable CAR targets in TNBC [9–17]. Among these, MSLN is particularly attractive due to its high expression in TNBC and limited expression in normal tissues [12, 18]. Preclinical studies have demonstrated the feasibility and partial efficacy of MSLN-specific CAR-T therapies in TNBC [11, 12]. However, their clinical translation has been constrained by key challenges, including suboptimal tumor trafficking, poor persistence in the hostile tumor microenvironment (TME), and severe manufacturing burdens associated with autologous CAR-T cell production [10, 11, 19]. Autologous approaches require individualized manufacturing from cancer patients, which is both time-consuming and expensive, and may not be feasible for patients with aggressive disease or insufficient T cell counts [20–23]. These limitations highlight the critical need for an effective and scalable off-the-shelf CAR-engineered cell therapy for TNBC.

We previously developed a clinically guided platform to generate allogeneic CAR-engineered invariant natural killer T cells (CAR-NKT) by integrating NKT TCR genes into human hematopoietic stem and progenitor cells (HSPCs), followed by ex vivo HSPC differentiation using a feeder-free culture system [24]. NKT cells represent a distinct innate-like T cell lineage that recognizes the non-polymorphic MHC class I-like molecule CD1d, enabling them to mount broad antitumor responses without triggering graft-versus-host disease (GvHD) [25–29]. Importantly, CAR-NKT cells exhibit several advantages over conventional CAR-T cells: they are capable of trafficking to solid tumors, reshaping the immunosuppressive TME, and executing cytotoxicity through both CAR and endogenous TCR pathways [24, 30–37]. These unique properties make CAR-NKT cells particularly well suited for developing allogeneic, off-the-shelf cell therapies targeting solid tumors such as TNBC.

In this study, we report the successful generation of allogeneic IL-15-enhanced MSLN-specific CAR-NKT (^Allo15^MCAR-NKT) cells with high yield and purity using our HSPC-engineered clinically guided culture platform [24]. We conducted comprehensive preclinical evaluations of these cells, including analyses using primary TNBC patient samples, in vitro functional assays, and in vivo orthotopic and metastatic human TNBC xenograft mouse models. We assessed their phenotypic characteristics, functional properties, antitumor efficacy, and safety profile. Notably, we demonstrate that these allogeneic CAR-NKT cells can be further engineered to eliminate HLA class I and II expression, rendering them resistant to host T cell-mediated allorejection while maintaining robust antitumor activity. These findings highlight the potential of ^Allo15^MCAR-NKT cells as a scalable, potent, and safe off-the-shelf immunotherapy for the treatment of TNBC.

## Methods

### Study approval

Animal studies were conducted under protocols approved by the UCLA Division of Laboratory Animal Medicine. Healthy donor PBMCs were obtained from the UCLA/CFAR Virology Core Laboratory and HemaCare under informed consent and in compliance with federal and state regulations; no identifying information was provided. Primary TNBC samples were collected at the Cedars-Sinai Medical Center from consented patients through an IRB-approved protocol (IRB #3032 Resistance Mechanisms in Breast Cancer) and processed.

### Mice

NOD.Cg-Prkdc^SCID^ Il2rg^tm1Wjl^ /SzJ (NOD/SCID/IL-2Rγ^−/−^, NSG) mice were purchased from The Jackson Laboratory (Strain #:005557; RRID: IMSR_JAX:005557), and maintained in the animal facilities of UCLA under the following housing conditions: temperature ranging from 68 °F to 79 °F, humidity maintained at 30–70%, a light cycle of On at 6:00 am and Off at 6:00 pm, and room pressure set to negative. 6–10 weeks old female mice were used for all experiments unless otherwise indicated. All animal experiments were approved by the Institutional Animal Care and Use Committee of UCLA (ARC-2013-054). All mice were bred and maintained under specific pathogen-free conditions, and all experiments were conducted in accordance with the animal care and use regulations of the Division of Laboratory Animal Medicine at the UCLA. Experimental mice were randomly assigned to treatment groups to avoid statistically significant differences in the baseline tumor burden.

### Media and reagents

The X-VIVO 15 Serum-Free Hematopoietic Cell Medium (cat. no. 04418Q) was purchased from Lonza. The StemSpan™ T Cell Generation Kit (cat. no. 09940), comprising the StemSpan™ SFEM II Medium (cat. no. 09605), the StemSpan™ Lymphoid Progenitor Expansion Supplement (cat. no. 09915), the StemSpan™ LPMS (cat. no. 09930), the StemSpan™ Lymphoid Progenitor Differentiation Coating Material (cat. no. 09925), and the ImmunoCult™ Human CD3/CD28/CD2 T Cell Activator (cat. no.10970), and MethoCult™ H4330 MethycelluloseBased Medium (cat. no. 04330) were purchased from StemCell Technologies. The CTS™ OpTmizer™ T-Cell Expansion SFM (no phenol red, bottle format, cat. no. A3705001), the RPMI 1640 cell culture medium (cat. no. MT10040CV), and the DMEM cell culture medium (cat. no. MT10013CV) were purchased from Thermo Fisher Scientific. The CryoStor ^®^ Cell Cryopreservation Media CS10 (cat. no. C2874) and Iscove’s Modified Dulbecco’s Medium (cat. no. I3390) was purchased from MilliporeSigma. The C10 medium was made of RPMI 1640 cell culture medium, supplemented with FBS (10% vol/vol), P/S/G (1% vol/vol), MEM NEAA (1% vol/vol), HEPES (10 mM), Sodium Pyruvate (1 mM), Beta-Mercaptoethanol (β-ME) (50 µM), and Normocin (100 µg/ml). The homemade D10 medium was made of DMEM supplemented with FBS (10% vol/vol), P/S/G (1% vol/vol), and Normocin (100 µg/ml). The homemade D10 medium was made of DMEM supplemented with FBS (10% vol/vol), P/S/G (1% vol/vol), and Normocin (100 µg/ml).

α-Galactosylceramide (αGC, KRN7000, cat. no. 867000) was purchased from Avanti Polar Lipids. Recombinant human IL-2 (cat. no. 200-02), IL-3 (cat. no. 200-03), IL-7 (cat. no. 200-07), IL-15 (cat. no. 200 − 15), IFN-γ (cat. no. 300-02), Flt3 ligand (Flt3L, cat. no. 300 − 19), macrophage colony stimulating factor (M-CSF, cat. no. 300 − 25), stem cell factor (SCF, cat. no. 300-07), and thrombopoietin (TPO, cat. no. 300 − 18) were purchased from Peprotech. Fetal Bovine Serum (FBS, lot no. 2087050) were purchased from Gibco and β-ME (cat. no. 1610710) were purchased from Bio-Rad. Penicillin Streptomycin-Glutamine (P/S/G, cat. no. 10-378-016), MEM nonessential amino acids (NEAA, cat. no. 11-140-050), HEPES Buffer Solution (cat. no. 15630080), and Sodium Pyruvate (cat. no. 11360070) were purchased from Gibco. Normocin was purchased from InvivoGen (cat. no. NC9390718).

### Lentiviral vectors

A parental lentivector, pMNDW, was utilized to construct the lentiviral vectors employed in this study [38, 39]. The 2 A sequences derived from foot-and-mouth disease virus (F2A), porcine teschovirus-1 (P2A), and thosea asigna virus (T2A) were used to link the inserted genes to achieve co-expression. The Lenti/iNKT-MCAR-IL-15 vector was generated by inserting into the pMNDW parental backbone a synthetic tetracistronic gene encoding human iNKT TCRα-F2A-iNKT TCRβ-P2A-MCAR-T2A-IL-15 (MCAR denotes an MSLN-specific CAR, and IL-15 represents the secreted form of human interleukin-15). The Lenti/iNKT-MCAR vector was generated by inserting into the pMNDW parental backbone a synthetic tricistronic gene encoding human iNKT TCRα-F2A-iNKT TCRβ-P2A-MCAR. The Lenti/iNKT-CAR19-IL-15 vector was generated by inserting into the pMNDW parental backbone a synthetic tetracistronic gene encoding human iNKT TCRα-F2A-iNKT TCRβ-P2A-CAR19-T2A-IL-15 (CAR19 denotes a CD19-specific CAR). The Lenti/iNKT-IL-15 vector was generated by inserting into the pMNDW parental backbone a synthetic tricistronic gene encoding human iNKT TCRα-F2A-iNKT TCRβ-P2A-IL-15. The Lenti/MCAR vector was generated by inserting into the pMNDW parental backbone a synthetic gene encoding MCAR. The Lenti/MCAR-IL-15 vector was generated by inserting into the pMNDW parental backbone a synthetic bicistronic gene encoding MCAR-F2A-IL-15. The Lenti/FG vector was generated by inserting into the pMNDW parental backbone a synthetic bicistronic gene encoding Fluc-P2A-EGFP. The Lenti/MSLN vector was generated by inserting into the pMNDW parental backbone a synthetic gene encoding human MSLN. All synthetic gene fragments were obtained from GenScript (Piscataway, NJ, USA) and Integrated DNA Technologies (IDT; Coralville, IA, USA). Lentiviral particles were generated utilizing HEK 293 T cells by employing a standardized transfection procedure with the Trans-IT-Lenti Transfection Reagent (Mirus Bio) [38, 39]. Subsequently, a concentration protocol was applied using Amicon TM Ultra Centrifugal Filter Units in accordance with the manufacturer’s specifications (MilliporeSigma).

### Stable cell lines

Human multiple myeloma cell line MM.1 S (cat. no. CRL-2974) and human TNBC cell lines HCC1806 (cat. no. CRL-2335) and MDA-MB-231 (cat. no. CRM-HTB-26) were purchased from the American Type Culture Collection (ATCC). To establish stable tumor cell lines that overexpress firefly luciferase and green fluorescent protein dual reporters (FG), the parental tumor cell lines were transduced with lentiviral vectors carrying the specific genes of interest (i.e., Lenti/FG). 72 h after lentiviral transduction, the cells underwent flow cytometry sorting to isolate the genetically modified cells (as identified as GFP^+^ cells) necessary for creating stable cell lines. The artificial antigen presenting cell line (aAPC) was generated by engineering the K562 human chronic myelogenous leukemia cell line (ATCC, cat. no. CCL-243) to overexpress human CD80/CD83/CD86/41BBL co-stimulatory receptors [24]. The aAPC-MSLN cell lines were generated by further engineering the parental aAPC line to overexpress human MSLN.

### Human CD34^+^ hematopoietic stem and progenitor cells (HSPCs) and periphery blood mononuclear cells (PBMCs)

Purified human CD34^+^ HSPCs derived from cord blood (CB) were purchased from HemaCare. Healthy donor PBMCs were provided by the UCLA/CFAR Virology Core Laboratory without identification information under federal and state regulations. Upon receipt, both HSPCs and PBMCs were promptly aliquoted and cryopreserved in liquid nitrogen for subsequent experimental use.

### Antibodies and flow cytometry

Fluorochrome-conjugated antibodies specific for human CD1d (Clone 51.1, PE-Cy7 or APC-conjugated, 1:50, cat. no. 350310 or 350308), CD3 (Clone HIT3a, Pacific Blue, PE, or PE-Cy7-conjugated, 1:500, cat. no. 300330, 300308, or 300316), CD4 (Clone OKT4, PE-Cy7, PerCP or FITC-conjugated, 1:500, cat. no. 317414, 317432 or 317408), CD8 (Clone SK1, PE, APC-Cy7, or APC-conjugated, 1:300, cat. no. 344706, 344714 or 344722), CD14 (Clone HCD14, Pacific Blue-conjugated, 1:100, cat. no. 367122), CD19 (Clone HIB19, APC-Cy7-conjugated, 1:200, cat. no. 302218), CD25 (Clone BC96, PE-conjugated, 1:100, cat. no. 302606), CD34 (Clone 581, PerCP-conjugated, 1:500, cat. no. 343520), CD31 (Clone WM59, FITC-conjugated, 1:100, cat. no. 989002), CD45 (Clone HI30, PerCP, FITC, or Pacific Blue-conjugated, 1:500, cat. no. 982318, 982316, or 982306), CD69 (Clone FN50, PE-Cy7 or PerCP-conjugated, 1:50, cat. no. 310912 or 310928), CD112 (Clone TX31, PE-conjugated, 1:250, cat. no. 337410), CD155 (Clone SKII.4, PE-Cy7-conjugated, 1:250, cat. no. 337614), CD11b (Clone ICRF44, FITC-conjugated, 1:500, cat. no. 982614), MICA/MICB (Clone 6D4, PE or APC-conjugated, 1:25, cat. no. 320906 or 320908), 41BBL (Clone 5F4, PE-conjugated, 1:500, cat. no. 311504), CD83 (Clone HB15e, APC-Cy7-conjugated, 1:500, cat. no. 305330), CD86 (Clone IT2.2, APC-conjugated, 1:500, cat. no. 305412), PD-1 (Clone A17188A, PE or FITC-conjugated, 1:25, cat. no. 379210 or 379206), TIM-3 (Clone A18087E, APC-conjugated, 1:25, cat. no. 364804), CTLA-4 (Clone L3D10, APC-conjugated, 1:50, cat. no. 369606), TIGIT (Clone A15153G, PE-conjugated, 1:50, cat. no. 372706), LAG-3 (Clone 7H2C65, PE-Cy7-conjugated, 1:25, cat. no. 369208), NKG2D (Clone 1D11, PE-Cy7-conjugated, 1:50, cat. no. 320812), DNAM-1 (Clone 11A8, APC-conjugated, 1:50, cat. no. 338312), NKp30 (Clone P30-15, APC-conjugated, 1:50, cat. no. 325210), NKp46 (Clone 9E2, PE-conjugated, 1:50, cat. no. 331908), CD158 or KIR2DL1/S1/S3/S5 (Clone HP-MA4, PE-Cy7-conjugated, 1:50, cat. no. 339512), IFN-γ (Clone B27, PE-Cy7-conjugated, 1:50, cat. no. 506518), Granzyme B (Clone QA16A02, APC-conjugated, 1:2000 or 1:5000, cat. no. 372204), Perforin (Clone dG9, PE-Cy7-conjugated, 1:50 or 1:100, cat. no. 308126), TNF-α (Clone MAb11, APC-conjugated, 1:4000, cat. no. 502912), IL-2 (Clone MQ117H12, APC-Cy7-conjugated, 1:50, cat. no. 500342), β2-microglobulin (B2M) (Clone 2M2, FITC or APC-conjugated, 1:2000, cat. no. 316304 or 316311), HLA-DR (Clone L243, APC-Cy7-conjugated, 1:200, cat. no. 307618), HLA-DR, DP, DQ (Clone Tü39, FITC-conjugated, 1:200 or 1:500, cat. no. 361706), TROP2 (Clone NY18, PE-conjugated, 1:200, cat. no. 363804), EGFR (Clone A21043C, FITC-conjugated, 1:200, cat. no. 386306), and EpCAM (Clone 9C4, PE or Pacific Blue-conjugated, 1:500, cat. no. 324206 or 324218) were purchased from BioLegend. Fluorochrome-conjugated antibodies specific for human iNKT TCR Vɑ24-Jβ18 (Clone 6B11, PE-conjugated, 1:20, cat. no. 552825) were purchased from BD Biosciences. Fluorochrome-conjugated antibodies specific for human fibroblast activation protein FAP (Clone 427819, PE-conjugated, 1:100, cat. no. FAB3715P), ULBP-1 (Clone 170818, PE-conjugated or unconjugated, 1:25, cat. no. FAB1380P or MAB1380), ULBP-2,5,6 (Clone 165903, APC-conjugated, 1:25, cat. no. FAB1298A), MSLN (Clone 420411, APC-conjugated, 1:20, cat. no. FAB32652A), and Nectin-4 (Clone 337516, PE-conjugated, 1:200, cat. no. FAB2659P), were purchased from R&D Systems. A goat anti-mouse IgG F(ab’)2 secondary antibody (cat. no. A-11001) was purchased from ThermoFisher. Fixable Viability Dye eFluor506 (e506, 1:500, cat. no. 65-0866-14) was purchased from Affymetrix eBioscience; mouse Fc Block (anti-mouse CD16/32, cat. no. 553141) was purchased from BD Biosciences; and human Fc Receptor Blocking Solution (TrueStain FcX) was purchased from BioLegend (cat. no. 422302). In our study, note the use of antibodies with identical clones but differing conjugated fluorochromes, with one typical antibody listed herein.

All FACS staining was performed following manufacturers’ provided protocols. Appropriate isotype staining controls were used for all staining procedures. Stained cells were analyzed using a MACSQuant Analyzer 10 flow cytometer (Miltenyi Biotech), following the manufacturer’s instructions. FlowJo software version 9 (BD Biosciences) was used for data analysis.

### Enzyme-linked immunosorbent cytokine assays (ELISAs)

The ELISAs for measuring cytokines or biomarkers were conducted according to a standard protocol provided by BD Biosciences. Samples were collected and analyzed to quantify cytokines or biomarkers. The capture and biotinylated antibodies used for cytokine detection were sourced from BD Biosciences, while the streptavidin-HRP conjugate was obtained from Invitrogen. Human and mouse cytokine standards were purchased from eBioscience, and the Tetramethylbenzidine (TMB) substrate was acquired from Thermo Scientific (cat. no. PI34021). Human IL-17 A ELISA Kit was purchased from Invitrogen (cat. no. BMS2017). Urea Nitrogen (BUN) Colorimetric Detection Kit was purchased from ThermoFisher Scientific (cat. no. EIABUN). Mouse AST ELISA kit was purchased from Abcam (cat. no. ab263882). Mouse ALT ELISA kit was purchased from Abcam (cat. no. ab282882). Mouse Bilirubin ELISA Kit was purchased from MyBioSource (cat. no. MBS3805359). Mouse Glutamate dehydrogenase (GLDH) ELISA Kit was purchased from MyBioSource (cat. no. MBS761948). Absorbance of the samples was measured at 450 nm using an Infinite M1000 microplate reader (Tecan).

### Generation of HSPC-engineered allogeneic/universal IL-15-enhanced MSLN-specific CAR-engineered NKT (^Allo/U15^MCAR-NKT) cells

^Allo15^MCAR-NKT, ^U15^MCAR-NKT, ^Allo^MCAR-NKT, ^Allo15^NKT, and ^Allo15^CAR19-NKT cells were generated by differentiating gene engineered human cord blood CD34^+^ HSPCs in a 5-stage clinically guided Ex Vivo HSPC-Derived NKT Cell Culture method. The complete methodology and step-by-step protocols have been described in detail in previously published studies [24, 40]. Here, we provide a summary of the key steps involved in the culture and generation of ^Allo/U15^MCAR-NKT and ^Allo^MCAR-NKT cells.

At Stage 0, the frozen stock of human CD34^+^ HSPCs was thawed and cultured in T cell X-VIVO 15 Serum-Free Hematopoietic Stem Cell Medium supplemented with human Flt3L (50 ng/ml), SCF (50 ng/ml), TPO (50 ng/ml), and IL-3 (20 ng/ml) for 24 h. Lentiviral transduction was subsequently carried out for an additional 24 h using the Lenti/iNKT-MCAR-IL15 or Lenti/iNKT-MCAR vectors. For ^U15^MCAR-NKT cell generation, HSPCs were further electroporated with a CRISPR-Cas9/B2M-CIITA-gRNAs complex, following an established protocol [33]. The gRNA sequences are CGCGAGCACAGCUAAGGCCA (*B2M*) and GAUAUUGGCAUAAGCCUCCC (*CIITA*). Both gene knockouts were performed simultaneously to achieve dual disruption of *B2M* and *CIIT*A. Following electroporation, the mixed CD34⁺ HSPC population was moved to the next stage of cell culture without further purification or sorting.

At Stage 1, gene-engineered HSPCs harvested were cultured in the feeder-free StemSpan™ SFEM II Medium supplemented with StemSpan™ Lymphoid Progenitor Expansion Supplement for 14 days. HSPCs were cultured in CELLSTAR^®^24-well Cell Culture Nontreated Multiwell Plates (VWR, cat. no. 82050-892). StemSpan™ Lymphoid Differentiation Coating Material (500 µl/well, diluted to a final concentration of 1X from a stock dilution of 100X), which includes immobilized Delta-like ligands to provide Notch signaling in the absence of feeder cells [41, 42], was applied to the plates and left for 2 h at room temperature or overnight at 4°C. Subsequently, 500 µl of the transfected CD34^+^ HSPC suspension, with a density of 2 × 10^4^ cells/ml, was added to each pre-coated well. Half of the medium in each well was removed and replaced with fresh medium twice per week.

At Stage 2, the Stage 1 cells were harvested and cultured in the feeder-free StemSpan™ SFEM II Medium supplemented with StemSpan™ Lymphoid Progenitor Maturation Supplement for ~ 7 days. StemSpan™ Lymphoid Differentiation Coating Material (1 ml/well, diluted to a final concentration of 1X) was applied to Non-Treated Falcon™ Polystyrene 6-well Microplates (Thermo Fisher Scientific, cat. no. 140675); 2 ml of the harvested Stage 1 cells, resuspended with a density of 1 × 10^5^ cells/ml, was added into each pre-coated well. The cell density was maintained at 1–2 × 10^6^ cells per well during the Stage 2 culturing. Cells were passaged 2–3 times per week with the addition of fresh medium for each passage.

At Stage 3, the Stage 2 cells were harvested and cultured in the feeder-free StemSpan™ SFEM II Medium supplemented with StemSpan™ Lymphoid Progenitor Maturation Supplement, CD3/CD28/CD2 T Cell Activator, and human recombinant IL-15 (20 ng/ml) for ~ 7 days. StemSpan™ Lymphoid Differentiation Coating Material (1 ml/well, diluted to a final concentration of 1X) was applied to Non-Treated Falcon™ Polystyrene 6-well Microplates (Thermo Fisher Scientific, cat. no. 08-772-49); 2 ml of the harvested Stage 2 cells, resuspended with a density of 5 × 10^5^ cells/ml, was added into each pre-coated well. The cell density was maintained at 1–2 × 10^6^ cells per well during the Stage 3 culturing. Cells were passaged 2–3 times per week with the addition of fresh medium for each passage.

At Stage 4, the Stage 3 cells were harvested and verified by flow cytometry to confirm their status as mature ^Allo15^MCAR-NKT cells or their derivatives; then the cells underwent expansion stage via an aAPC-based expansion. aAPC-MSLN cells were irradiated at 10,000 rads using a Rad Source RS-2000 X-Ray Irradiator (Rad Source Technologies). The Stage 3 mature ^Allo/U15^MCAR-NKT cells and derivatives were co-cultured with the irradiated aAPC-MSLN cells (with a ratio of 1:1). The cells were resuspended in expansion medium (the CTS™ OpTmizer™ T-Cell Expansion Serum Free Medium (Thermo Fisher Scientific), or the homemade C10 medium) supplemented with human IL-7 (10 ng/ml) and IL-15 (10 ng/ml) at a density of 0.5-1 × 10^6^ cells/ml; 2 ml cell suspension was seeded into each well of the Corning™ Costar™ Flat Bottom Cell Culture 6-well Plates. The cell density was maintained at 0.5-1 × 10^6^ cells/ml during the expansion stage. Cells were passaged 2–3 times per week with the addition of fresh medium for each passage. The expanded ^Allo/U15^MCAR-NKT cells were aliquoted and cryopreserved in CryoStor^®^ Cell Cryopreservation Media CS10 using a Thermo Scientific™ CryoMed™ Controlled-Rate Freezer 7450 (Thermo scientific) for stock.

Of note, at the research scale, we typically generated ^Allo15^MCAR-NKT cells starting from 10^4^ CD34⁺ HSPCs and successfully produced over 10^10^ mature cells. Cell cultures were maintained using either 150 mm cell culture dishes (Thermo Fisher Scientific) or G-Rex 6 M well plates (Wilson Wolf) to support large-scale expansion.

### Generation of PBMC-derived conventional αβ T cells

PBMCs from healthy donors were used to generate conventional αβ T cells (hereafter referred to as T cells). T cell activation was achieved using one of two methods: (1) stimulation with Dynabead™ Human T-Activator CD3/CD28 (Thermo Fisher Scientific, Cat. No. 11131D) according to the manufacturer’s instructions, or (2) plate-bound activation. For the latter, non-treated 24-well tissue culture plates (Corning, Cat. No. 3738) were coated with Ultra-LEAF™ purified anti-human CD3 antibody (Clone OKT3, BioLegend, cat. no. 317325) at 1 µg/ml (500 µl/well) for 2 h at room temperature or overnight at 4 °C. PBMCs were then resuspended in C10 medium supplemented with 1 µg/ml Ultra-LEAF™ purified anti-human CD28 antibody (Clone CD28.2, BioLegend, cat. no. 302933) and 30 ng/ml IL-2, and seeded into the pre-coated plates at a density of 1 × 10^6^ cells/ml (1 ml/well). Following activation, cells were maintained in C10 medium supplemented with 20 ng/ml IL-2 and cultured for 2–3 weeks. T cells generated by both methods were subsequently tested in the in vitro tumor cell killing assays and showed comparable efficacy, with neither demonstrating tumor cell killing activity.

### Generation of MSLN-specific CAR-engineered conventional αβ T (MCAR-T) cells

PBMCs from healthy donors were utilized to generate conventional MCAR-T cells. To produce these cells, non-treated tissue culture 24-well plates (Corning, cat. no. 3738) were coated with Ultra-LEAF™ Purified Anti-Human CD3 Antibody (Clone OKT3, BioLegend) at 1 µg/ml (500 µl/well), at room temperature for 2 h or at 4 °C overnight. PBMCs were resuspended in the C10 medium supplemented with 1 µg/ml Ultra-LEAF™ Purified Anti-Human CD28 Antibody (Clone CD28.2, BioLegend) and 30 ng/ml IL-2, followed by seeding in the pre-coated plates at 1 × 10^6^ cells/ml (1 ml/well). After 2 days, the cells were transduced with either Lenti/MCAR viruses for a period of 24 h. The conventional MCAR-T cells were expanded for about 2 weeks in C10 medium and then cryopreserved for future applications.

### Generation of PBMC-derived IL-15-engineered MSLN-specific CAR-NKT (^PBMC15^MCAR-NKT) cells

Healthy donor PBMCs were sorted with MACS via Anti-iNKT Microbeads (Miltenyi Biotech, cat. no. 130-094-842) labeling to enrich NKT cells, following the manufacturer’s instructions. The enriched NKT cells were mixed with donor-matched irradiated αGC/PBMCs at a ratio of 1:1–1:2, followed by culturing in C10 medium supplemented with 10 ng/ml IL-7 and IL-15. On day 3, NKT cells were transduced with Lenti/MCAR-IL15 viruses. The resulting CAR-NKT cells were expanded for about 2 weeks in C10 medium supplemented with 10 ng/ml IL-7 and IL-15 and cryopreserved for future use.

### In vitro tumor cell killing assay

Human tumor cells (e.g., MM.1 S-FG and HCC1806-FG; 1 × 10^4^ cells per well in 96-well plate) were co-cultured with the indicated therapeutic cells (i.e., T, MCAR-T, and ^Allo15^MCAR-NKT cells) in Corning 96-well clear bottom black plates for 24 h in C10 medium. The effector cell to target cell (E: T) ratio is indicated in the figure legends. At the end of culture, viable tumor cells were quantified by adding D-luciferin (150 µg/ml; Fisher Scientific, cat. no. 50-209-8110) to cell cultures, followed by the measurement of luciferase activity using an Infinite M1000 microplate reader (Tecan). To test NK receptor-mediated tumor cell killing, 10 µg/ ml Ultra-LEAF™ purified anti-human NKG2D (Clone 1D11, BioLegend, cat. no. 320813) or anti-human DNAM-1 antibody (Clone 11A8, BioLegend, cat. no. 338302) was added to co-cultures to investigate the tumor cell killing mechanism by ^Allo15^MCAR-NKT cells, and LEAF™ purified mouse lgG2b κ isotype control antibody (Clone MG2b-57, BioLegend, cat. no. 401202) was included as an isotype control.

In vitro long-term tumor cell killing assay.

A total of 1 $\:\times\:$ 10^4^ non-engineered tumor cells (e.g., HCC1806 cells; referred to as stimulator cells) was co-cultured with 2 $\:\times\:$ 10^5^ effector cells in a Corning 96-well clear bottom black plate in C10 medium. Cultures were supplemented with a dose of 1 $\:\times\:$ 10^4^ stimulator cells every 2 days. 24 h prior to luminescent tumor killing readout, stimulator cells were substituted with 1 $\:\times\:$ 10^4^ of FG-engineered tumor cells (e.g., HCC1806-FG cells; referred to as indicator cells). To quantify the remaining live indicator cells, 100mL of D-luciferin (10 mg/mL) was added to cell cultures on the day of imaging and the luciferase activities were measured through readout with an Infinite M1000 microplate reader (Tecan).

### In vitro 3D tumor organoid targeting assay

Healthy donor PBMC-derived, M2-polarized macrophages or MDSCs were used in this assay. PBMCs were resuspended in serum-free RPMI 1640 medium (Corning Cellgro, cat. no. 10-040-CV) at 1 × 10^7^ cells/ml, plated in 10 cm dishes (10–15 ml per dish), and incubated at 37 °C with 5% CO_2_ for 1 h. Non-adherent cells were removed, and adherent monocytes were utilized to generate M2-polarized macrophages or MDSCs. To generate M2-polarized macrophages, monocytes were cultured in C10 medium supplemented with recombinant human M-CSF (10 ng/ml, Peprotech, cat. no. 300 − 25) for 6 days. On day 6, macrophages were detached using 0.25% Trypsin/EDTA (Gibco, cat. no. 25200-056), collected, and reseeded in 6- or 12-well plates (0.5-1 × 10^6^ cells/ml) for another 48 h with recombinant human IL-4 (10 ng/mL, Peprotech, cat. no. 214 − 14) and IL-13 (10 ng/ml, Peprotech, cat. no. 214 − 13) to induce M2 polarization. To generate MDSCs, monocytes were cultured in C10 medium supplemented with human GM-CSF and IL-6 (10 ng/mL) for 6 days.

To generate tumor organoids, a 1:1 mixture of 1 × 10^5^ HCC1806-FG tumor cells and 1 × 10^5^ M2 macrophages/MDSCs were resuspended in C10 medium at a concentration of 1 × 10^5^ cells/µl. Cell aggregates were prepared by dispensing 5–10 µl of the cell suspension onto microporous membrane inserts (EMD Millipore, cat. no. PICM0RG50) placed in 6-well plates containing 1 ml of C10 medium per well [43, 44]. After a 2-day incubation period to allow organoid formation, 1 × 10^6^ therapeutic cells (i.e., MCAR-T or ^Allo15^MCAR-NKT cells) were resuspended in 100 µl of C10 medium and added on top of each organoid. Co-culture was maintained for 24 h. Following the co-culture period, organoids were mechanically dissociated with a 1 ml pipette and passed through a 70-µm nylon strainer to generate single-cell suspensions for downstream flow cytometry analysis.

### In vitro assays using primary TNBC patient samples

In one assay, the primary TNBC patient samples were analyzed for tumor cell phenotype and the TME composition using flow cytometry. TNBC tumor cells identified as CD45^−^CD31^−^FAP (fibroblast activation protein)^−^ cells [45, 46], T cells were identified as CD45^high^CD3^+^ cells, CD4 T cells were identified as CD45^high^CD3^+^CD4^+^ T cells, CD8 T cells were identified as CD45^high^CD3^+^CD8^+^ T cells, B cells were identified as CD45^high^CD19^+^ cells, NK cells were identified as CD45^high^CD56^+^CD3^−^ cells, monocytes were identified as CD45^high^CD11b^+^CD14^+^ cells, tumor-associated macrophages (TAMs) were identified as HLA-DR^high^CD206^high^ monocytes, and myeloid-derived suppressor cells (MDSCs) were identified as HLA-DR^low^CD206^low^ monocytes. Surface expression of CAR targets and NK ligands on tumor or/and immune cells were also analyzed using flow cytometry.

The clinical patient survival data were obtained from the Tumor Immune Dysfunction and Exclusion (TIDE) database, illustrating the association between MSLN expression in tumors and patient survival in a breast cancer cohort (Prediction of Clinical Outcomes from Genomic Profiles: GSE20486, *n* = 97). The expression data of CAR target antigens (i.e., MSLN, TROP2, EGFR, and Nectin-4) on TNBC tumor cells and normal tissues were derived from previously published studies [11, 47–52].

**Fig. 1:**
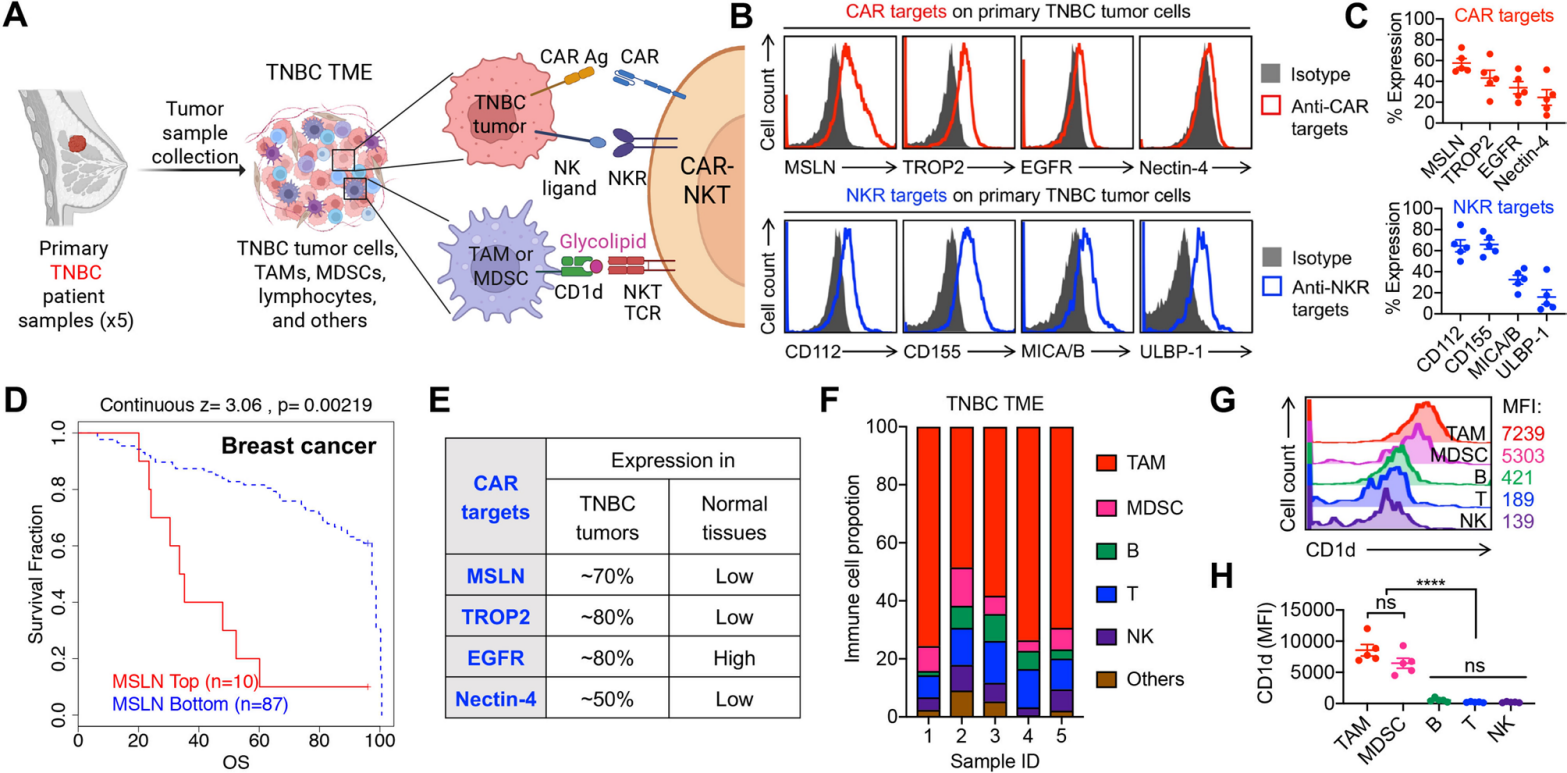
Primary metastatic TNBC patient sample profiling highlights the therapeutic potential of CAR-NKT cells. (**A**) Diagram showing the primary metastatic TNBC patient sample collection, the TME composition, and the tumor and TME targeting mechanisms of CAR-NKT cells. TNBC, triple negative breast cancer; TME, tumor microenvironment; TAM, tumor associated macrophages; MDSC, myeloid-derived suppressor cell; NKR, NK receptor; Ag, antigen. (**B**) FACS analyses of CAR targets (e.g., MSLN, TROP2, EGFR, and Nectin-4) and NKR targets (e.g., CD112, CD155, MICA/B, and ULBP-1) expression on tumor cells from TNBC patient samples. MSLN, mesothelin. (**C**) Quantification of (B) (*n* = 5). (**D**) Clinical data correlation studies. Kaplan-Meier plots are presented, showing the association between *MSLN* expression in tumor and survival of cancer patients in a breast cancer cohort (Prediction of Clinical Outcomes from Genomic Profiles [PRECOG]: GSE20486, *n* = 97). (**E**) Table showing the CAR antigen expressions on TNBC tumors and normal tissues. (**F**) Immune cell composition of 5 TNBC patients. (**G**) FACS analyses of CD1d expression on the indicated immune cells. (**H**) Quantification of (G) (*n* = 5). Representative of 5 experiments. Data are presented as the mean ± SEM. ns, not significant, ****p < 0.0001, by one-way ANOVA (H)

In another assay, the primary TNBC patient samples were used to study tumor cell killing by ^Allo15^MCAR-NKT cells. Tumor cells were pre-sorted using a Human Tumor Cell Isolation Kit (Miltenyi Biotec, cat. no. 130-108-339), followed by co-culturing with various therapeutic cells (E: T ratio 1:1) in C10 medium in Corning 96-well Round Bottom Cell Culture plates for 24 h. At the end of culture, cells were collected and live TNBC tumor cells (identified as CD45^−^CD3^−^6B11^−^ cells) was analyzed using flow cytometry. A total of 5 primary OC patient samples were included in this assay.

In another assay, the primary TNBC patient samples were used to study the TME targeting by ^Allo15^MCAR-NKT cells. Patient samples were directly co-cultured with ^Allo15^MCAR-NKT cells (ratio 1:1) in C10 medium in Corning 96-well Round Bottom Cell Culture plates for 24 h. At the end of culture, cells were collected, and the TNBC TME targeting of ^Allo15^MCAR-NKT cells was assessed using flow cytometry by quantifying live human TAM (identified as 6B11^−^CD45^+^CD14^+^CD11b^+^HLA-DR^high^CD206^high^ cells), MDSCs (identified as 6B11^−^CD45^+^CD14^+^CD11b^+^HLA-DR^low^CD206^low^ cells), T cells (identified as 6B11^−^CD45^+^CD3^+^ cells), B cells (identified as 6B11^−^CD45^+^CD3^−^CD19^+^ cells or 6B11^−^CD45^+^CD3^−^CD20^+^ cells), and NK cells (identified as 6B11^−^CD45^+^CD3^−^CD56^+^ cells). A total of 5 primary TNBC patient samples were included in this assay.

### In vitro mixed lymphocyte reaction (MLR) assay

PBMCs of multiple healthy donors were used as responders, to study the T cell-mediated allorejection of ^Allo15^MCAR-NKT and ^U15^MCAR-NKT cells as stimulators (irradiated at 2,500 rads). MCAR-T cells were included as stimulator controls. Stimulators (5 × 10^5^ cells/well) and responders (2 × 10^4^ cells/well) were co-cultured in 96-well round bottom plates in C10 medium for 4 days; the cell culture supernatants were then collected to measure IFN-γ production using ELISA.

### In vivo bioluminescence live animal imaging (BLI)

BLI was conducted using the Spectral Advanced Molecular Imaging (AMI) HTX system (Spectral Instrument Imaging). Live animal images were captured 5 min after intraperitoneal (i.p.) administration of D-Luciferin, with doses of 1 mg/mouse for tumor cell (e.g., HCC1806-FG cell) visualization. For tissue imaging, experimental mice received an i.p. injection of D-Luciferin with doses of 10 mg/mouse. Mice were then euthanized, and tissues were collected for BLI. The imaging data were processed and analyzed using AURA imaging software (Spectral Instrument Imaging, version 3.2.0).

### In vivo antitumor efficacy study: HCC1806-FG human orthotopic TNBC xenograft NSG mouse model

On Day 0, NSG mice received orthotopic inoculation of human TNBC tumor cells (HCC1806-FG, 5 × 10^5^ or 3 × 10^6^ cells per mouse). The number of tumor cells used for implantation was selected based on preliminary optimization experiments to ensure consistent and measurable tumor growth within a defined timeframe. This cell dose reliably produces tumors of appropriate size and progression dynamics, allowing for accurate assessment of therapeutic efficacy while avoiding excessive tumor burden that could confound interpretation of treatment effects.

**Fig. 2:**
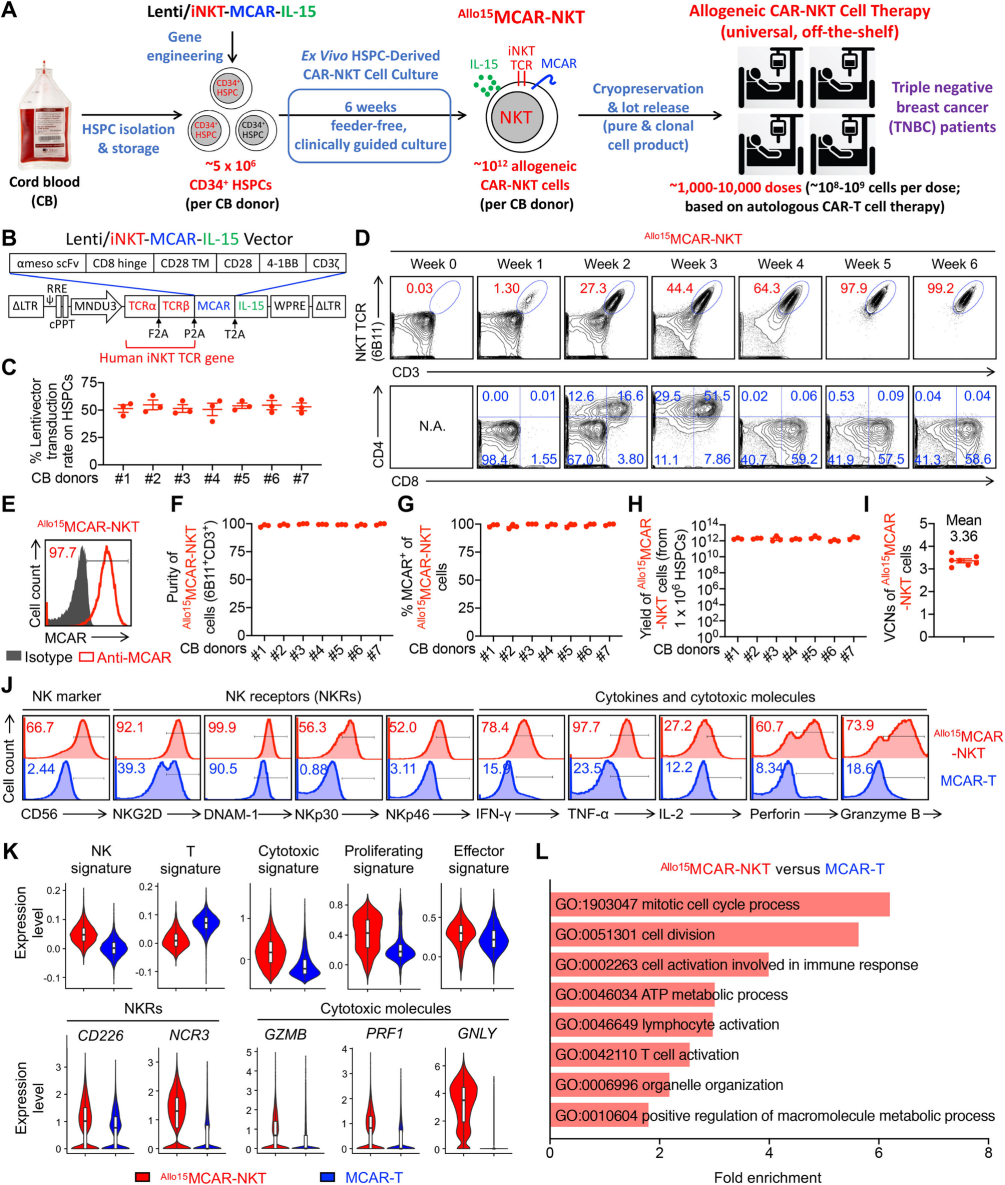
Allogeneic stem cell-derived MCAR-NKT cells are generated using a clinically guided culture method and display prominent NK-like features and potent cytotoxic activity. (**A**) Schematics showing the generation of ^Allo15^MCAR-NKT cells. CB, cord blood; HSPC, hematopoietic stem and progenitor cells; Lenti/iNKT-MCAR-IL-15, lentiviral vector encoding a pair of iNKT TCR α and β chains, a mesothelin-directed CAR, and a human soluble IL-15. (**B**) Schematics showing the design of Lenti/iNKT-MCAR-IL-15 lentivector. ΔLTR, self-inactivating long terminal repeats; MNDU3, internal promoter derived from the MND retroviral LTR U3 region; φ, packaging sequence; RRE, rev-responsive element; cPPT, central polypurine tract; WPRE, woodchuck hepatitis virus posttranscriptional regulatory element; F2A, foot-and-mouth disease virus 2 A; P2A, porcine teschovirus-1 2 A; T2A, thosea asigna virus 2 A. (**C**) FACS analyses of Lenti/iNKT-MCAR-IL-15 transduction rate on CD34^+^ HSPCs at 72 h after lentivector transduction (*n* = 3). 7 different cord blood donors were tested. (**D**) FACS monitoring of the generation of ^Allo15^MCAR-NKT cells during the 6-week culture. iNKT TCR was stained using a 6B11 monoclonal antibody. (**E**) FACS detection of CAR expression on ^Allo15^MCAR-NKT cells. (**F**) Quantification of ^Allo15^MCAR-NKT cell purity (identified as 6B11^+^ and CD3^+^) (*n* = 3). 7 different cord blood donors were tested. (**G**) Quantification of CAR^+^ proportion of ^Allo15^MCAR-NKT cell (*n* = 3). 7 different cord blood donors were tested. (**H**) Yield of ^Allo15^MCAR-NKT cells (*n* = 3). 7 different cord blood donors were tested. (**I**) VCN measurement of ^Allo15^MCAR-NKT cells (*n* = 7). VCN, vector copy number. (**J**) FACS detection of NK marker and NK receptor (NKR) expression, as well as intracellular cytokine and cytotoxic molecule production of ^Allo15^MCAR-NKT and conventional MCAR-T cells. (**K**) Violin plots showing the expression distribution of the indicated gene signatures in ^Allo15^MCAR-NKT and conventional MCAR-T cells. (**L**) Pathway analyses of differentiated expressed genes comparing ^Allo15^MCAR-NKT with conventional MCAR-T cells. GO, Gene ontology ID. Representative of 3 (A-I) and 1 (J-L) experiments. Data are presented as the mean ± SEM

On Day 4, the experimental mice received peritumor (p.t.) injection of vehicle (100 µl PBS per mouse), ^Allo15^MCAR-NKT cells (5 × 10^6^ cells in 100 µl PBS per mouse), conventional MCAR-T cells (5 × 10^6^ cells in 100 µl PBS per mouse), ^Allo15^NKT cells (5 × 10^6^ cells in 100 µl PBS per mouse), or ^Allo15^CAR19-NKT cells (5 × 10^6^ cells in 100 µl PBS per mouse). Notably, MCAR-T cells were enriched to > 95% MCAR⁺ purity prior to injection. Over the experiment, mice were monitored for survival and their tumor load were measured using BLI and tumor size measurement. At the study endpoint, experimental mice were euthanized, and survival data were collected to generate Kaplan–Meier survival curves. To evaluate the GvHD risk, experimental mice were assessed for clinical GvHD scores on days 40 and 50. A score ranging from 0 to 2 was assigned for each clinical GvHD sign, which includes body weight, activity, posture, skin thickening, diarrhea, and dishevelment [53]. In addition, on Day 1, 20, and 50, serum samples were collected from the experimental mice, and the organ damage biomarkers were measured using ELISA. Urea Nitrogen (BUN) Colorimetric Detection Kit was purchased from ThermoFisher Scientific (cat. no. EIABUN). Mouse AST ELISA kit was purchased from Abcam (cat. no. ab263882). Mouse ALT ELISA kit was purchased from Abcam (cat. no. ab282882). Mouse Bilirubin ELISA Kit was purchased from MyBioSource (cat. no. MBS3805359). Mouse Glutamate dehydrogenase (GLDH) ELISA Kit was purchased from MyBioSource (cat. no. MBS761948).

### In vivo antitumor efficacy study: MDA-MB-231-FG human orthotopic TNBC xenograft NSG mouse model

On Day 0, NSG mice received orthotopic inoculation of human TNBC tumor cells (MDA-MB-231-FG, 3 × 10^6^ cells per mouse). On Day 4, the experimental mice received peritumor (p.t.) injection of vehicle (100 µl PBS per mouse), ^Allo15^MCAR-NKT cells (5 × 10^6^ cells in 100 µl PBS per mouse), or conventional MCAR-T cells (5 × 10^6^ cells in 100 µl PBS per mouse). Notably, MCAR-T cells were enriched to > 95% MCAR⁺ purity prior to injection. Over the experiment, mice were monitored for survival and their tumor load were measured using BLI.

### In vivo antitumor efficacy study: HCC1806-FG human metastatic TNBC xenograft NSG mouse model

On Day 0, 5 × 10^5^ HCC1806-FG cells were intravenously injected into the NSG mice to establish a metastatic TNBC model. On Day 4, vehicle (100 µl PBS per mouse), ^Allo15^MCAR-NKT cells (10 × 10^6^ cells in 100 µl PBS per mouse), ^U15^MCAR-NKT cells (10 × 10^6^ cells in 100 µl PBS per mouse), or conventional MCAR-T (10 × 10^6^ cells in 100 µl PBS per mouse) cells were administered intravenously. Notably, MCAR-T cells were enriched to > 95% MCAR⁺ purity prior to injection. Over the experiment, mice were monitored for survival and their tumor load were measured using BLI. At the study endpoint, experimental mice were euthanized, and survival data were collected to generate Kaplan-Meier survival curves.

**Fig. 3:**
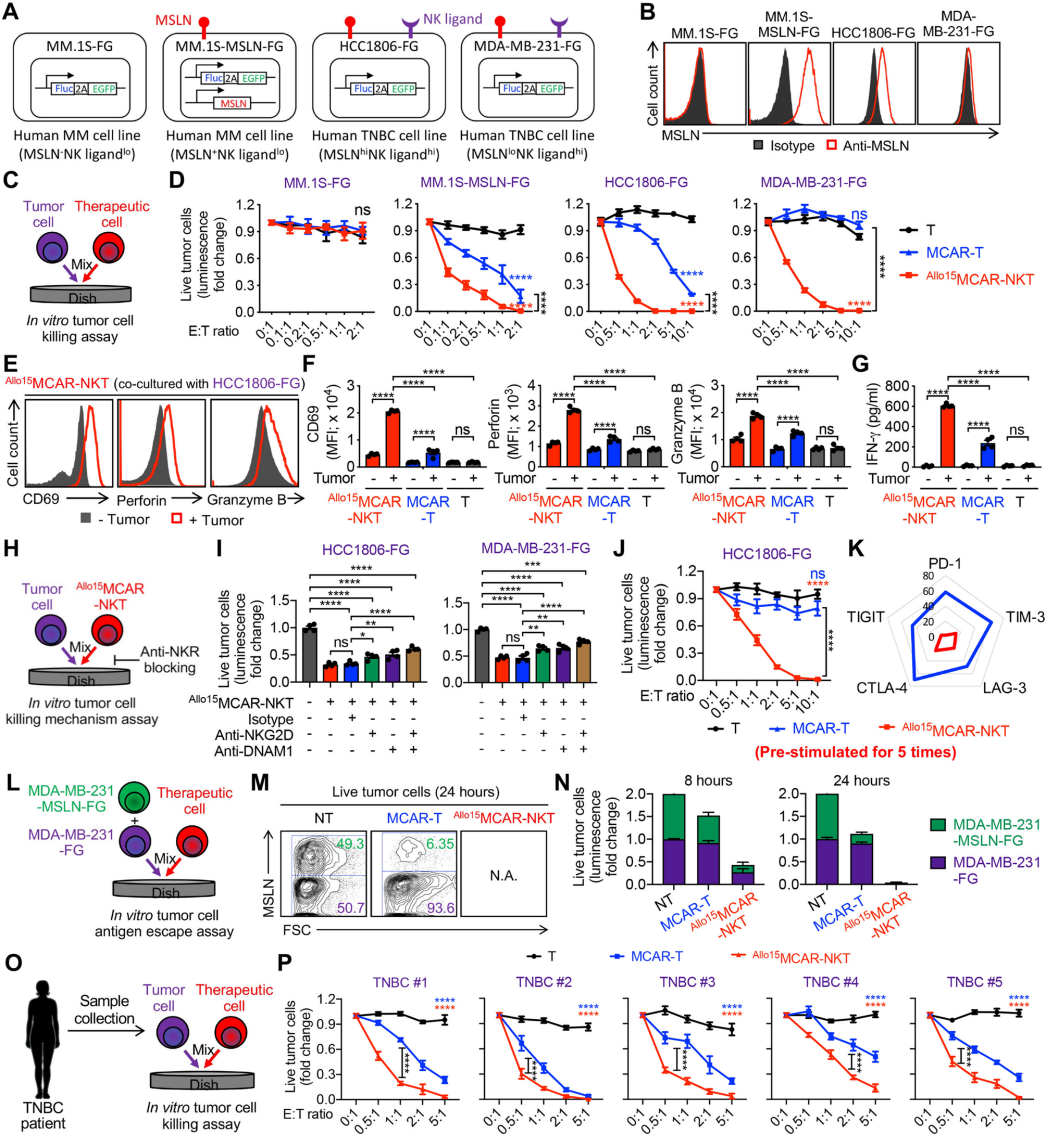
Allogeneic MCAR-NKT cells kill TNBC tumor cells with superior antitumor efficacy and multifaceted killing mechanisms. (**A**-**D**) Studying the in vitro antitumor efficacy of ^Allo15^MCAR-NKT cells against human MM and TNBC cell lines. MCAR-T cells and non-MCAR-engineered PBMC-derived T cells were included as therapeutic cell controls. (**A**) Schematics showing the indicated human MM and TNBC cell lines. MM.1 S-FG, MM.1 S cell engineered to overexpress FG; MM.1 S-MSLN-FG, MM.1 S-FG cell engineered to overexpress MSLN; HCC1806-FG, HCC1806 cell engineered to overexpress FG; MDA-MB-231-FG, MDA-MB-231 cell engineered to overexpress FG. (**B**) FACS detection of MSLN expression on the indicated tumor cells. (**C**) Experimental design. (**D**) Tumor cell killing data at 24 h (*n* = 4). (**E**-**G**) Studying the expression of effector molecules of ^Allo15^MCAR-NKT cells. (**E**) FACS detection of surface CD69 as well as intracellular Perforin and Granzyme B in the therapeutic cells. (**F**) Quantification of (**E**) (*n* = 4). (**G**) ELISA analyses of IFN-γ production in the therapeutic cells (*n* = 4). (**H**-**I**) Studying the tumor cell killing mechanisms of ^Allo15^MCAR-NKT cells mediated by NKRs (**i**.**e**., NKG2D and DNAM-1). (**H**) Experimental design. (**I**) Tumor cell killing data at 24 h (**E**: T ratio = 0.5:1; *n* = 4). (**J**) Studying the long-term tumor cell killing of ^Allo15^MCAR-NKT cells after repeated tumor challenge (*n* = 4). HCC1806-FG tumor cells were added every 48 h for 5 times. (**K**) Radar plots showing the expression of immune checkpoint molecules on ^Allo15^MCAR-NKT cells. Data represent percent-positive cells as measured by flow cytometry. (**L**-**N**) Studying the tumor antigen escape targeting of ^Allo15^MCAR-NKT cells. (**L**) Experimental design. (**M**) FACS analyses of remaining live MSLN^+^ and MSLN^−^ tumor cells at 24 h. (**N**) Quantification of remaining live MDA-MB-231-MSLN-FG and MDA-MB-231-FG cells at 24 h. The percentages of MDA-MB-231-MSLN-FG and MDA-MB-231-FG cells were recorded, and the fold changes were calculated by normalizing to the NT group. (**O**-**P**) Studying the TNBC tumor cell killing capacity of ^Allo15^MCAR-NKT cells against primary TNBC patient samples. (**O**) Experimental design. (**P**) Tumor cell killing data at 24 h (*n* = 4). Representative of 3 experiments. Data are presented as the mean ± SEM. ns, not significant, *p < 0.05, **p < 0.01, ***p < 0.001, ****p < 0.0001 by two-way ANOVA (**D**, **J**, and **P**), or by one-way ANOVA (**F**, **G**, and **I**)

**Fig. 4:**
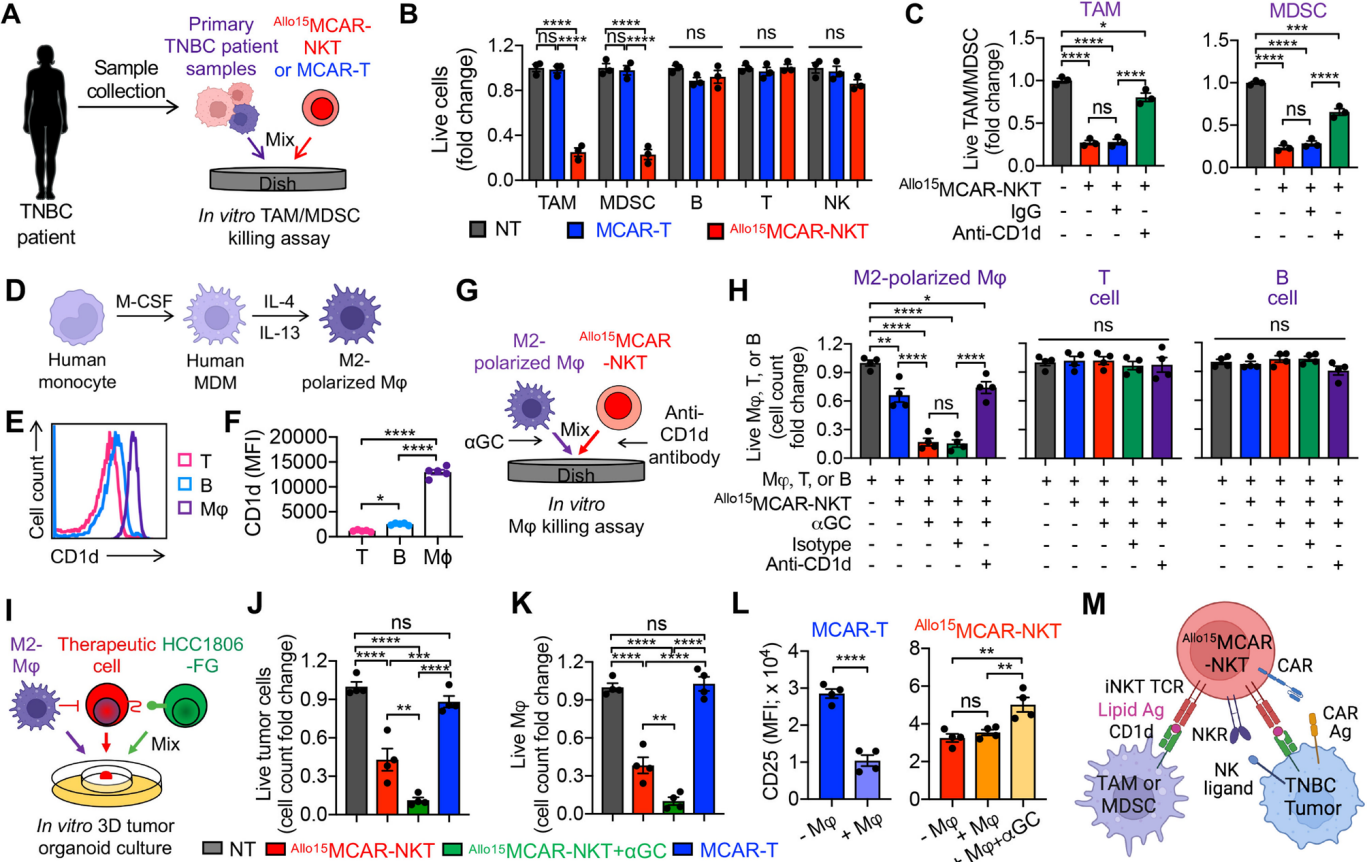
Allogeneic MCAR-NKT cells target the TNBC TME by killing the CD1d^+^ immunosuppressive cells. (**A**-**B**) Studying primary metastatic TNBC-derived TAM/MDSC targeting by ^Allo15^MCAR-NKT cells. (**A**) Experimental design. (**B**) FACS analyses of live immune cells targeted by ^Allo15^MCAR-NKT cells at 24 h (*n* = 3). The percentage of the indicated immune cells among total CD45^+^CAR^−^ immune cells was recorded, and the fold change was calculated by normalizing to the NT group. (**C**) Studying CD1d-mediated TAM/MDSC targeting by ^Allo15^MCAR-NKT cells at 24 h (*n* = 3). Anti-CD1d antibody was added into the co-culture to block CD1d/NKT TCR recognition. (**D**-**F**) In vitro generation and polarization of human monocyte-derived M2 macrophages. (**D**) Experimental design. M-CSF, macrophage colony-stimulating factor; MDM, monocyte-derived macrophage; Mφ, macrophage. (**E**) FACS detection of CD1d on M2-polarized macrophages. Healthy donor PBMC-derived **T** and B cells were included as staining controls. (**F**) Quantification of (**E**) (*n* = 5). (**G**-**H**) Studying M2-polarized macrophage targeting by ^Allo15^MCAR-NKT cells. (**G**) Experimental design. (**H**) FACS analyses of live macrophages at 24 h after co-culturing with ^Allo15^MCAR-NKT cells (*n* = 4). Live cells were identified as e506^−^CD14^+^CD11b^+^ cells. An anti-CD1d antibody was added into the coculture to block CD1d/iNKT TCR recognition. Healthy donor PBMC-derived **T** and B cells were included as target cell controls. (**I**-**L**) Studying TAM targeting by ^Allo15^MCAR-NKT cells using an in vitro 3D tumor organoid culture. (**I**) Experimental design. (**J**) Tumor killing data at 48 h (*n* = 4). (**K**) TAM killing data at 48 h (*n* = 4). (**L**) FACS analyses of surface markers (**i**.**e**., CD25) of ^Allo15^MCAR-NKT cells. (**M**) Diagram showing the tumor-targeting and TAM-targeting mechanisms of ^Allo15^MCAR-NKT cells. Representative of 3 experiments. Data are presented as the mean ± SEM. ns, not significant, *p < 0.05, **p < 0.01, ***p < 0.001, ****p < 0.0001 by Student’s *t* test (L left), or by one-way ANOVA (B, C, H, J, K, and L right)

### Single cell RNA sequencing (scRNA-seq)

Freshly collected samples were immediately delivered to the UCLA TCGB Core for library construction and scRNA-seq. Cells were quantified using a Cell Countess II automated cell counter (Invitrogen/Thermo Fisher Scientific). A total of 10,000 cells from each experimental group were loaded on the Chromium platform (10X Genomics), and libraries were constructed using the Chromium Next GEM Single Cell 3’ Kit and the Chromium Next GEMChip G Single.

Cell Kit (10X Genomics), according to themanufacturer’s instructions. Library quality was assessed using the D1000 ScreenTape on a 4200 TapeStation System (Agilent Technologies). Libraries were sequenced on an Illumina NovaSeq using the NovaSeq S4 Reagent Kit (100 cycles; Illumina). AddModuleScore was used to calculate module scores of each list of gene signatures, and FeaturePlot function was used to visualize the expression of each signature in the UMAP plots [54, 55].

### Statistics

Graphpad Prism 9 software (Graphpad) was used for statistical data analysis. Student’s two-tailed t test was used for pairwise comparisons. Ordinary 1-way ANOVA followed by Tukey’s or Dunnett’s multiple comparisons test was used for multiple comparisons. Log rank (Mantel-Cox) test adjusted for multiple comparisons was used for Meier survival curves analysis. Data are presented as the mean ± SEM, unless otherwise indicated. In all figures and figure legends, “n” represents the number of samples or animals used in the indicated experiments. A *P* value of less than 0.05 was considered significant. ns, not significant.

## Results

### Primary TNBC patient sample profiling highlights the therapeutic potential of CAR-NKT cells

One of the major challenges in developing effective cell-based immunotherapies for TNBC is the lack of a comprehensive profile of immune target biomarkers expressed on tumor cells and within the tumor microenvironment (TME) throughout disease progression [10, 56]. To address this gap, we conducted an immunophenotypic analysis of primary TNBC tumor cells and their corresponding TME using flow cytometry (Fig. 1A). Primary tumor samples were obtained from five TNBC patients, and both tumor cells and infiltrating immune populations were profiled (Table S1). Flow cytometric analysis enabled the identification of multiple cell types, including tumor cells, tumor-associated macrophages (TAMs), myeloid-derived suppressor cells (MDSCs), T cells, B cells, and NK cells (Fig. S1A). All five primary tumor samples exhibited high levels of EpCAM expression, confirming their epithelial identity (Fig. S1B) [57].

We first evaluated the expression of tumor-associated antigens that are potential CAR targets in TNBC, including MSLN, TROP2, EGFR, and Nectin-4. These markers exhibited heterogeneous expression across patient samples (Fig. 1B and C). Among them, MSLN showed consistently high expression levels on TNBC tumor cells and was associated with poorer overall survival, indicating its role in disease progression (Fig. 1B–1D). Importantly, MSLN expression was low in normal tissues, highlighting it as a promising and tumor-selective CAR target (Fig. 1E) [18]. Based on these findings, MSLN was selected for subsequent CAR engineering efforts. In addition to conventional CAR targets, we also observed robust and stable expression of natural killer receptor (NKR) ligands, such as MIC-A/B and ULBPs (NKG2D ligands), and CD112/CD155 (DNAM-1 ligands), on TNBC tumor cells across all samples, suggesting the potential for NKR-mediated recognition and killing (Fig. 1B and C).

Flow cytometry of the TME revealed a significant enrichment of immunosuppressive myeloid populations, particularly TAMs and MDSCs, alongside other immune cells such as T cells, B cells, and NK cells (Fig. 1F). Notably, a biomarker screening identified high expression of CD1d, a non-polymorphic, MHC class I-like molecule, on TAMs and MDSCs, but not on other immune subsets (Fig. 1G and H). As CD1d is the canonical ligand for the invariant TCR expressed by NKT cells, these findings suggest that CD1d^hi^ TAMs and MDSCs are natural targets for NKT cell-mediated cytotoxicity [45, 58–60].

Consistent with this, we detected the presence of NKT cells within the TNBC TME, with frequencies ranging from 0.1 to 1% across samples (Fig. S1C), indicating that NKT cells are capable of infiltrating the TME and may contribute to modulating the tumor immune environment. NKT cells uniquely combine TCR-mediated recognition of CD1d-expressing cells with the expression of multiple activating NKRs, such as NKG2D and DNAM-1, which are capable of recognizing ligands commonly upregulated on TNBC tumor cells [25, 26, 28, 32, 38]. Furthermore, NKT cells can be engineered to express additional CARs, such as MSLN-targeting CARs, further enhancing their tumor-targeting capabilities. In summary, our immune profiling of primary TNBC samples has revealed that TNBC is particularly susceptible to CAR-NKT cell therapy (Fig. 1A). This vulnerability arises from the concurrent expression of tumor-associated CAR targets, NKR ligands, and CD1d-expressing immunosuppressive myeloid cells in the TME (Fig. 1A). These findings support a triple-targeting strategy, leveraging the TCR, CAR, and NKR axes of NKT cells, as a promising immunotherapeutic approach for overcoming the challenges of TNBC treatment.

### Allogeneic stem cell-derived MCAR-NKT cells are generated using a clinically guided culture method

NKT cells are rare in human peripheral blood (0.001-1%), posing a significant challenge for generating clinically relevant quantities of allogeneic NKT cells suitable for CAR engineering [31, 61, 62]. Moreover, allogeneic NKT cells expanded from blood may be contaminated with bystander conventional αβ T cells, raising the risk of GvHD [63–65]. To overcome these limitations, we developed a stem cell-based approach and a clinically guided culture method to produce “off-the-shelf” CAR-NKT cells to treat TNBC with high yield, purity, and functional robustness (Fig. 2A) [24, 40].

**Fig. 5:**
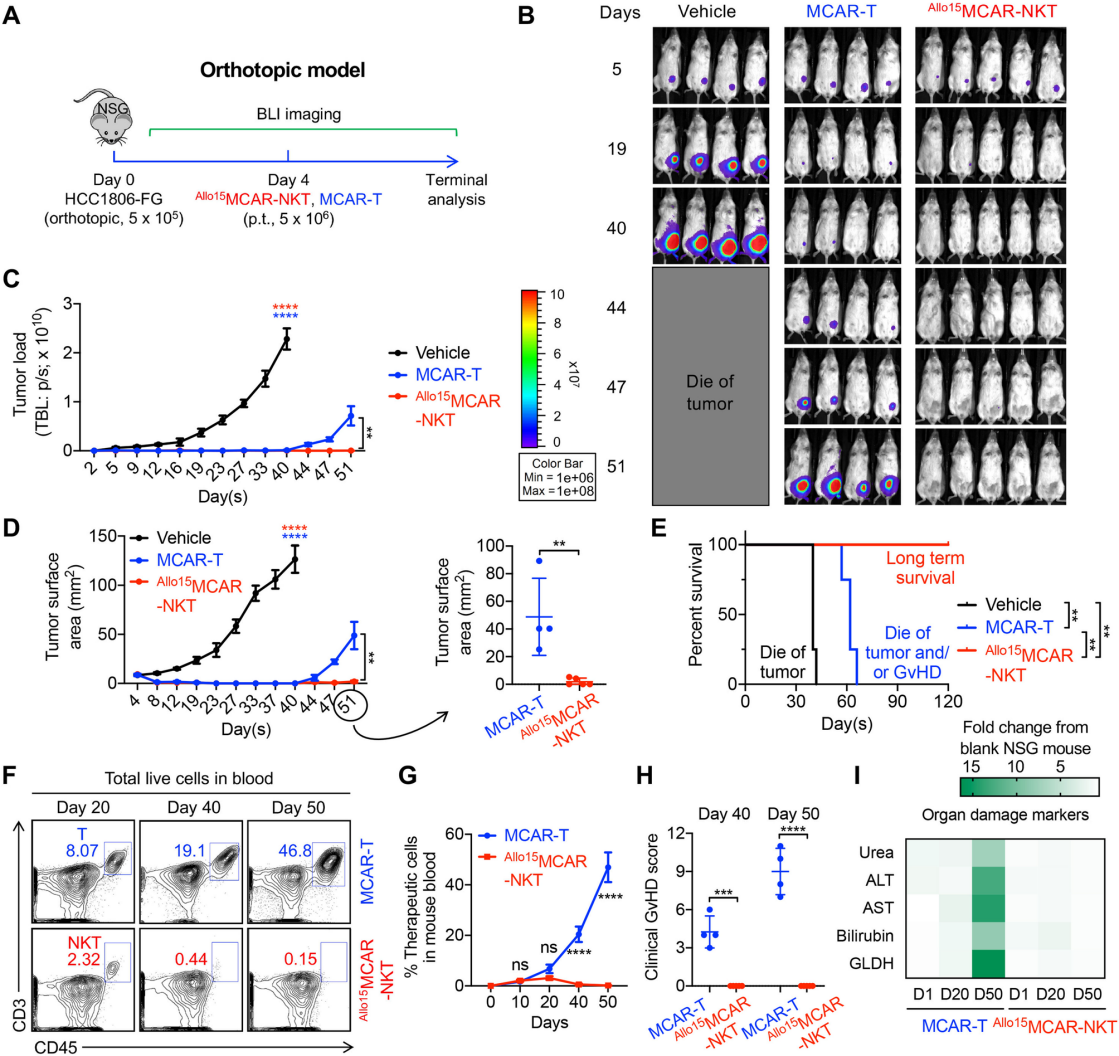
Allogeneic MCAR-NKT cells demonstrate superior antitumor efficacy and safety in a human TNBC orthotopic xenograft model. (**A**-**E**) Studying the in vivo antitumor efficacy of ^Allo15^MCAR-NKT cells in an orthotopic HCC1806-FG human xenograft NSG mouse model. (**A**) Experimental design. BLI, live animal bioluminescence imaging. (**B**) BLI images measuring tumor loads in experimental mice over time. (**C**) Quantification of (**B**) (*n* = 4–5). (**D**) Tumor size measurements in experimental mice over time (*n* = 4–5). (E) Kaplan–Meier survival curves (*n* = 4–5). (**F**-**I**) Studying the in vivo safety of ^Allo15^MCAR-NKT cells. (**F**) FACS analyses of CD3^+^CD45^+^ double positive T cells in mouse blood post injection of therapeutic cells. (**G**) Quantification of (**F**) (*n* = 4–5). (**H**) Clinical GvHD scores recorded on day 40 and 50 (*n* = 4–5). (**I**) Measurement of organ damage markers (Urea, ALT, AST, Bilirubin, and GLDH) in mouse serum. ALT, Alanine transaminase; AST, Aspartate Transaminase; GLDH, Glutamate Dehydrogenase. Representative of 3 experiments. Data are presented as the mean ± SEM. ns, not significant, **p < 0.01, ***p < 0.001, ****p < 0.0001 by Student’s *t* test (C right, and D right), by one-way ANOVA (C left, D left, G, and H) or by log rank (Mantel-Cox) test adjusted for multiple comparisons (E)

We constructed a lentiviral vector, Lenti/iNKT-MCAR-IL-15, enabling the co-expression of a human invariant NKT TCR, a mesothelin-specific third-generation CAR (MCAR), and secreted human IL-15 (Fig. 2B). The MCAR construct incorporates a clinically validated SS1 anti-mesothelin scFv and a 28-BBz intracellular signaling domain (Fig. 2B) [66, 67]. Human CD34⁺ HSPCs from cord blood (CB) were transduced with this vector and differentiated into CAR-NKT cells using our feeder-free, 6-week Ex Vivo HSPC-Derived CAR-NKT Cell Culture system [24, 40].

CD34⁺ HSPCs from seven independent CB donors (obtained from commercial vendors such as HemaCare) were successfully transduced and utilized to generate allogeneic IL-15-enhanced MCAR-NKT (^Allo15^MCAR-NKT) cells (Fig. 2C and S2A). By the end of the differentiation process, the engineered HSPCs gave rise to a homogeneous population of ^Allo15^MCAR-NKT cells, with > 97% co-expression of NKT TCR, CD3, and MCAR (Fig. 2D and E). These cells followed a developmental trajectory consistent with natural NKT cell ontogeny, transitioning from CD4^−^CD8^−^ double-negative (DN) to CD4^+^CD8^+^ double-positive (DP), and subsequently maturing into CD8^+^ single-positive (SP) or DN subtypes (Fig. 2D) [39, 68, 69]. The final cell population was predominantly CD4^−^CD8^+/−^, a phenotype known to be pro-inflammatory and cytotoxic, thus favorable for cancer immunotherapy [28, 65, 70, 71].

We next evaluated IL-15 expression and production by ^Allo15^MCAR-NKT cells. Upon stimulation with α-GalCer (αGC), a potent NKT cell agonist, ^Allo15^MCAR-NKT cells produced robust levels of IL-15, confirming successful transgenic expression and functional secretion of IL-15 (Fig. S2B). To assess the potential for autonomous or dysregulated proliferation, we conducted an in vitro cell proliferation assay in the absence of exogenous cytokine support (Fig. S2C). ^Allo15^MCAR-NKT cells failed to survive or expand under cytokine-free conditions, indicating that despite their ability to secrete IL-15, they remain dependent on exogenous IL-7 and IL-15 for survival and proliferation. These results suggest a low risk of cytokine-driven, uncontrolled cell growth (Fig. S2D).

Importantly, the ^Allo15^MCAR-NKT cell products were free of contaminating αβ T cells, eliminating the risk of GvHD (Fig. 2D and F). Across all CB donors, cell products consistently demonstrated > 97% purity and clonality (Fig. 2F and G). Calculated theoretical yield estimates indicated that from a single quality CB donor (~ 5 × 10^6^ CD34⁺ HSPCs), approximately 10^12 Allo15^MCAR-NKT cells could be generated, that is sufficient for formulating an estimated 1,000–10,000 clinical doses (at 10^8^-10^9^ cells per dose) (Fig. 2H) [6, 72, 73]. Vector copy number (VCN) analysis revealed an average of 3–4 copies per ^Allo15^MCAR-NKT cell, within the clinically accepted threshold (< 5 copies/cell), supporting the safety of the product with minimal risk of insertional mutagenesis (Fig. 2I) [6, 72, 73].

In summary, we established a robust, clinically relevant platform for generating high-yield, high-purity ^Allo15^MCAR-NKT cells from CB HSPCs. These cells will be evaluated for their therapeutic potential against TNBC and represent a promising candidate for off-the-shelf allogeneic cell therapy.

### Allogeneic MCAR-NKT cells display prominent NK-like features and potent cytotoxic activity

We next evaluated the phenotype and functional attributes of ^Allo15^MCAR-NKT cells and performed a direct comparison with conventional MCAR-engineered T cells (MCAR-T), which were generated from healthy donor peripheral blood mononuclear cells (PBMCs) and served as a benchmark control (Fig. S2E-S2I). While MCAR-T cells typically exhibited CAR expression in over 70% of the population, variability in manufacturing and purification steps can lead to inconsistencies in CAR expression across production batches (Fig. S2F and S2H). In contrast, ^Allo15^MCAR-NKT cells consistently achieved near-uniform (> 97%) MCAR expression, eliminating the need for additional purification or CAR-enrichment procedures (Fig. 2F and G). This uniformity underscores their off-the-shelf therapeutic potential and facilitates robust quality control during manufacturing. In addition, neither ^Allo15^MCAR-NKT cells nor conventional MCAR-T cells expressed MSLN, similar to their non-CAR-engineered counterparts, thereby avoiding self-fratricide mediated by the MSLN-specific CAR (Fig. S2I).

Flow cytometry analysis revealed that ^Allo15^MCAR-NKT cells possessed a phenotype closely aligned with endogenous human NKT cells, enriched for NK-like characteristics and cytotoxic functionality. Compared to conventional MCAR-T cells, ^Allo15^MCAR-NKT cells expressed higher levels of NK-associated markers such as CD56, along with activating NK receptors (NKRs) including NKG2D, DNAM-1, NKp30, and NKp46 (Fig. 2J). Notably, they lacked expression of inhibitory NKRs, such as killer-cell immunoglobulin-like receptors (KIRs) (Fig. S2J and S2K). Functionally, ^Allo15^MCAR-NKT cells demonstrated enhanced secretion of key effector cytokines (e.g., IFN-γ, TNF-α, and IL-2) and elevated levels of cytolytic molecules such as Perforin and Granzyme B (Fig. 2J). These phenotypic and functional features, consistent with their CD8 SP or DN lineage (Fig. 2D), support their potential utility in cancer immunotherapy by combining potent cytotoxicity with broad receptor-mediated tumor recognition.

To characterize the genomic and molecular features of ^Allo15^MCAR-NKT cells, we performed single-cell RNA sequencing (scRNA-seq) analysis, using conventional MCAR-T cells as a benchmark control. ^Allo15^MCAR-NKT cells displayed a hybrid T/NK cell transcriptional profile, marked by a higher NK cell gene signature and a comparatively lower T cell signature relative to MCAR-T cells (Fig. 2K). ^Allo15^MCAR-NKT cells also exhibited elevated expression of genes associated with proliferation and effector function (Fig. 2K). Notably, ^Allo15^MCAR-NKT cells upregulated NKR genes such as *CD226* and *NCR3*, as well as genes encoding the cytotoxic effector molecules including Perforin and Granzyme B, consistent with flow cytometry results (Fig. 2J and K). In addition, they expressed high levels of the NKT-lineage transcription factor *ZBTB16* (encoding PLZF) and *BCL11B* (encoding B-cell lymphoma/leukemia 11B), as well as the Th1-associated transcription factor *TBX21* (encoding T-bet) (Fig. S3A and S3B) [74]. Pathway analysis further revealed that, compared to MCAR-T cells, ^Allo15^MCAR-NKT cells were enriched for gene signatures associated with cell cycle progression, proliferation, activation, and metabolism (Fig. 2L). Collectively, these findings indicate that ^Allo15^MCAR-NKT cells possess potent T/NK-like cytotoxic characteristics, supporting their therapeutic potential for antitumor applications.

In addition to conventional MCAR-T cells, we conducted a side-by-side comparison of ^Allo15^MCAR-NKT cells with healthy donor PBMC-derived IL-15-enhanced MCAR-NKT (^PBMC15^MCAR-NKT) cells using scRNA-seq (Figure S3C). Both cell types expressed key NKT-lineage and Th1-associated transcription factors, including *ZBTB16*, *BCL11B*, and *TBX21*, indicating their shared identity as NKT cells with Th1-like functional potential (Figure S3D). Notably, compared to ^PBMC15^MCAR-NKT cells, ^Allo15^MCAR-NKT cells exhibited enhanced expression of NK-associated markers and reduced expression of conventional T cell–associated genes, along with higher proliferative and effector signatures but lower expression of cytotoxic gene programs (Figure S3E). Pathway analysis further revealed that ^Allo15^MCAR-NKT cells upregulated pathways involved in peptide synthesis, ATP biosynthesis, ribosome biogenesis, lymphocyte activation, and NK cell–mediated cytotoxicity (Figure S3F). These results suggest that ^Allo15^MCAR-NKT cells possess a metabolically active, proliferative, and NK-like effector phenotype that may contribute to their unique therapeutic potential.

### Allogeneic MCAR-NKT cells kill TNBC tumor cells through superior antitumor efficacy and multifaceted killing mechanisms

To evaluate the in vitro antitumor capacity of ^Allo15^MCAR-NKT cells against TNBC, we conducted a series of tumor cell killing assays using both established TNBC cell lines and primary tumor cells derived from patients. These tumor models differed in antigen expression, molecular characteristics, and genetic background, providing a comprehensive platform to assess the breadth of antitumor activity of ^Allo15^MCAR-NKT cells.

We first examined both CAR- and NKR-mediated cytotoxicity using four tumor cell lines: two NK cell-resistant multiple myeloma (MM) lines, MM.1 S (MSLN^−^) and MM.1 S-MSLN (engineered to overexpress MSLN), and two NK-sensitive TNBC cell lines, HCC1806 (MSLN^high^) and MDA-MB-231 (MSLN^low^) (Fig. 3A and B). All tumor lines were engineered to co-express firefly luciferase and green fluorescence protein dual reporters (FG) for quantifiable tracking via bioluminescence and flow cytometry (Fig. 3A).

**Fig. 6:**
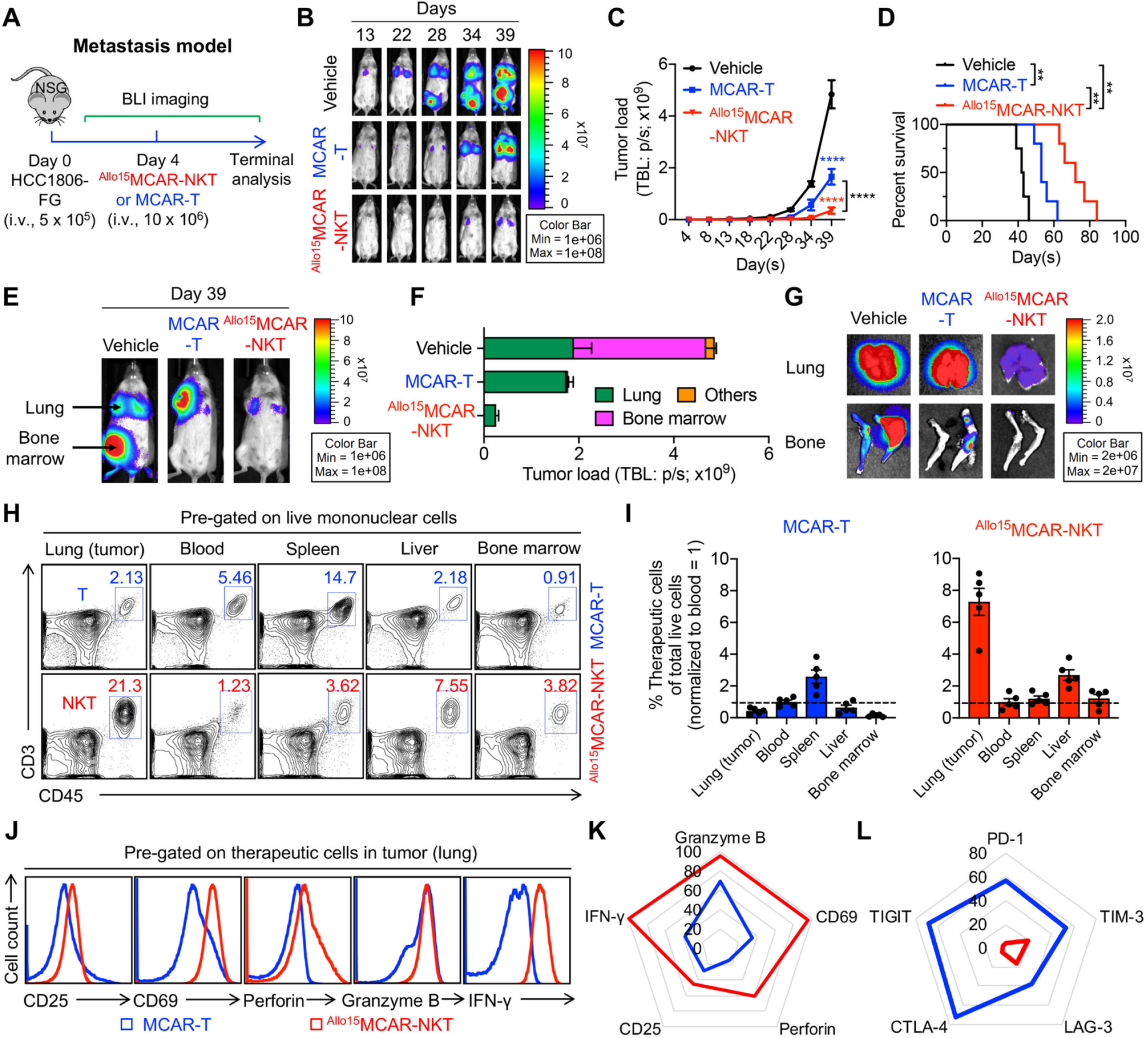
Allogeneic MCAR-NKT cells demonstrate superior antitumor efficacy with strong effector function and low exhaustion in a human metastatic TNBC xenograft model. (**A**-**G**) Studying the in vivo antitumor efficacy of ^Allo15^MCAR-NKT cells in a metastatic HCC1806-FG human xenograft NSG mouse model. (**A**) Experimental design. (**B**) BLI images measuring tumor loads in experimental mice over time. (**C**) Quantification of (**B**) (*n* = 4–5). (**D**) Kaplan–Meier survival curves (*n* = 4–5). (**E**) BLI images showing the biodistribution of HCC1806-FG cells in a representative experimental mouse on day 39. (**F**) Quantification of tumor loads in the indicated organs (*n* = 4–5). (**G**) BLI images showing the biodistribution of HCC1806-FG tumor cells in the indicated organs. (**H**-**I**) Studying the in vivo distribution of ^Allo15^MCAR-NKT cells on day 30. (**H**) FACS analyses of CD3^+^CD45^+^ double positive human T cells in different organs post injection of the therapeutic cells. (**I**) Quantification of (**H**) (*n* = 5). (**J**-**L**) Studying the in vivo profile of ^Allo15^MCAR-NKT cells. (**J**) FACS analyses of activation and effector markers of the therapeutic cells collected from the lungs. (**K**-**L**) Radar plots showing the expression of activation and effector markers (**K**) and immune checkpoint molecules (**L**) on the therapeutic cells. Data represent percent-positive cells as measured by flow cytometry. Representative of 3 experiments. Data are presented as the mean ± SEM. **p < 0.01, ****p < 0.0001 by two-way ANOVA (C), or by log rank (Mantel-Cox) test adjusted for multiple comparisons (D)

Three therapeutic immune cell groups were tested: (1) ^Allo15^MCAR-NKT cells, (2) conventional PBMC-derived MCAR-T cells, and (3) untransduced T cells from healthy donors (Fig. 3C). Within 24 h, unmodified T cells failed to exhibit cytotoxicity toward any of the tumor cell lines. As expected, MCAR-T cells efficiently lysed MSLN^+^ tumor cells but showed no activity against MSLN^−^ targets, confirming the CAR-dependent nature of their killing (Fig. 3D). In contrast, ^Allo15^MCAR-NKT cells robustly killed both MSLN^+^ and MSLN^−^ TNBC cells, indicating the use of both CAR-dependent and CAR-independent mechanisms (Fig. 3D).

Phenotypic analysis revealed that ^Allo15^MCAR-NKT cells displayed heightened activation (CD69 upregulation) and produced significantly greater levels of effector cytokines (e.g., IFN-γ) and cytotoxic molecules (e.g., Perforin and Granzyme B) than MCAR-T cells (Fig. 3E – 3G). Importantly, ^Allo15^MCAR-NKT cells showed antigen-dependent cytokine secretion, producing high levels of IFN-γ, TNF-α, IL-2, and IL-15 in response to MSLN-positive HCC1806-FG tumor cells—consistent with antigen-specific activation and cytolytic function (Fig. S4A and S4B). In contrast, co-culture with MSLN-negative target cells, including MM.1 S-FG and normal human cells (i.e., B cells), resulted in minimal cytokine production, indicating functional specificity and a low risk of off-target activation (Fig. S4A and S4B).

To dissect the CAR-independent cytotoxic mechanism, we performed NKR-blocking assays using neutralizing antibodies against NKG2D and DNAM-1 (Fig. 3H). Blockade of these receptors significantly impaired the cytotoxicity of ^Allo15^MCAR-NKT cells, confirming a reliance on NKR-mediated tumor recognition (Fig. 3I). Therefore, ^Allo15^MCAR-NKT cells utilize a dual targeting mechanism mediated by both the MCAR and endogenous NKRs to effectively target TNBC. To further dissect the contribution of each targeting axis, we incorporated two additional therapeutic cell controls: non–CAR-engineered allogeneic IL-15-enhanced NKT (^Allo15^NKT) cells and allogeneic IL-15-enhanced CD19-specific CAR-NKT (^Allo15^CAR19-NKT) cells (Fig. S5A). In the in vitro tumor cell killing assay, none of the tumor lines mentioned above expressed CD19, allowing us to evaluate CAR-independent functions of ^Allo15^NKT and ^Allo15^CAR19-NKT cells (Fig. S5B). Both cell types exhibited comparable tumor-killing profiles: no cytotoxicity against MM.1 S-FG or MM.1 S-MSLN-FG cells, but robust killing of HCC1806-FG and MDA-MB-231-FG cells (Fig. S5C). These findings indicate that ^Allo15^NKT and ^Allo15^CAR19-NKT cells mediate potent tumor cell killing via CAR-independent, NKR-mediated mechanisms, as demonstrated by the NKR blocking assay in which tumor cell killing was markedly reduced upon NKR (i.e., NKG2D and DNAM-1) blockade (Fig. S5D and S5E). In contrast, ^Allo15^MCAR-NKT cells showed robust killing of MM.1 S-MSLN-FG cells, which express the MSLN target antigen, and enhanced killing of MSLN-positive HCC1806-FG cells (Fig. S5C). Together, these results indicate that ^Allo15^MCAR-NKT cells possess dual-targeting capability via MCAR and NKR, whereas ^Allo15^NKT and ^Allo15^CAR19-NKT cells mediate killing primarily through NKR recognition.

We then investigated the long-term killing potential of ^Allo15^MCAR-NKT cells using repeated tumor challenge assays (Fig. 3J). Remarkably, ^Allo15^MCAR-NKT cells sustained superior cytolytic activity over five rounds of tumor exposure, whereas MCAR-T cells exhibited diminished function (Fig. 3J). This durable killing of ^Allo15^MCAR-NKT cells was associated with low expression of exhaustion markers, including PD-1, CTLA-4, TIM-3, LAG-3, and TIGIT (Fig. 3K), along with sustained high levels of IFN-γ production (Fig. S6A), suggesting enhanced persistence and resistance to functional decline. Furthermore, in a parallel long-term tumor killing assay, ^Allo15^MCAR-NKT cells outperformed non–IL-15-engineered allogeneic MCAR-NKT (^Allo^MCAR-NKT) cells, maintaining robust and sustained cytotoxic activity for up to 10 rounds of tumor rechallenge (Fig. S6B-S6G). These findings demonstrate that IL-15 expression enhances the durability, persistence, and antitumor efficacy of allogeneic MCAR-NKT cells under repeated antigen stimulation [75, 76].

To directly model tumor antigen escape, a known limitation of conventional CAR-T therapies [77, 78], we generated a mixed population of MDA-MB-231 tumor cells comprising both MSLN^+^ (engineered) and MSLN^−^ populations (Fig. 3L and S6H-S6K). While MCAR-T cells selectively eliminated MSLN^+^ tumor cells, they spared MSLN^−^ cells (Fig. 3M and N). In contrast, ^Allo15^MCAR-NKT cells efficiently eliminated both populations, highlighting their capacity to overcome tumor heterogeneity and antigen escape through multifaceted targeting mechanisms (Fig. 3M and N). While this in vitro model provides a useful proxy for tumor heterogeneity and antigen escape, we acknowledge its limitations in fully recapitulating dynamic antigen loss in vivo. Future studies will incorporate long-term in vivo models to more accurately evaluate this phenomenon.

Lastly, we extended these findings to primary patient-derived TNBC tumor cells (Fig. 3O). Across five independent samples, which varied in their expression of MSLN and NKR ligands (Fig. 1B and C), ^Allo15^MCAR-NKT cells consistently outperformed MCAR-T cells in cytotoxicity assays (Fig. 3P). To further dissect antigen-specific versus antigen-independent killing, tumor cells were FACS-sorted into MSLN^high^ and MSLN^low^ populations and co-cultured with the two therapeutic cell products (Fig. S6L and S6M). MCAR-T cells effectively eliminated MSLN^high^ tumor cells but spared MSLN^low^ tumor cells, confirming their reliance on CAR antigen recognition for cytotoxicity (Fig. S6N). In contrast, ^Allo15^MCAR-NKT cells exhibited cytotoxicity against both MSLN^high^ and MSLN^low^ populations, with enhanced killing of MSLN^high^ tumor cells (Fig. S6N). These results indicate that ^Allo15^MCAR-NKT cells mediate both CAR-dependent and CAR-independent cytotoxicity, likely via their native NKR pathways. This multiple-targeting capability enables effective tumor cell killing across heterogeneous antigen landscapes and supports their potential utility in addressing interpatient variability and antigen escape in TNBC.

Collectively, these results demonstrate that ^Allo15^MCAR-NKT cells exhibit potent and durable tumor cell killing through CAR and NKR pathways. Their ability to target antigen-diverse and antigen-low tumor populations supports their potential as a next-generation immunotherapeutic strategy for treating heterogeneous and aggressive tumors such as TNBC.

### Allogeneic MCAR-NKT cells target the TNBC TME by killing the CD1d^+^ immunosuppressive cells

The immunosuppressive TME represents a major barrier to effective cancer immunotherapy, particularly in solid tumors such as TNBC [79–82]. TNBC is characterized by a highly immunosuppressive TME that promotes tumor progression, facilitates immune evasion, and contributes to resistance to immunotherapeutic approaches [10, 83, 84]. Immune profiling of primary TNBC samples has revealed that TAMs and MDSCs comprise over 50% of the total immune cell population, underscoring the critical importance of targeting these immunosuppressive components to improve therapeutic outcomes (Fig. 1F).

TAMs and MDSCs in the TNBC TME express high levels of CD1d, a nonpolymorphic MHC class I-like molecule that is the natural ligand for the invariant TCR expressed by NKT cells (Fig. 1G and H) [31, 71]. Thus, these cells are promising targets for elimination by ^Allo15^MCAR-NKT cells (Fig. 1A). To investigate this therapeutic potential, we evaluated the interaction of ^Allo15^MCAR-NKT cells with TNBC TME components using three complementary models: primary patient-derived TNBC samples (Fig. 4A–4C), in vitro-generated healthy donor PBMC-derived macrophages and MDSCs (Fig. 4D–4H and S7A-S7E), and 3D TNBC tumor organoids (Fig. 4I–4L and S7F-S7I).

**Fig. 7:**
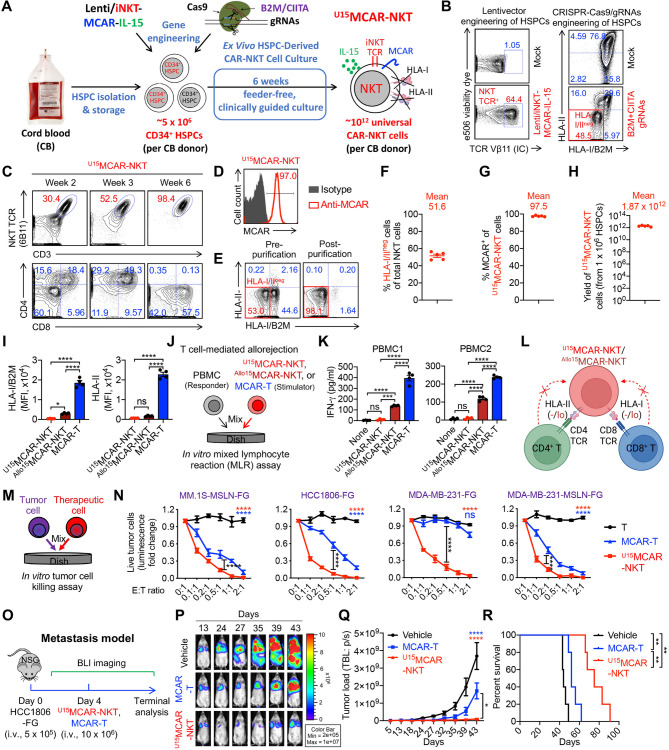
HLA-I/II-ablated universal MCAR-NKT cells resist host T cell-mediated allorejection while preserving potent antitumor efficacy. (**A**) Schematics showing the generation of HLA-ablated universal IL-15-enhanced MCAR-NKT (^U15^MCAR-NKT) cells. (**B**) FACS analyses of NKT TCR and HLA-I/II expression in CD34^+^ HSPCs at 72 h after lentivector transduction. (**C**) FACS monitoring of the generation of ^U15^MCAR-NKT cells during the 6-week culture. (**D**) FACS analyses of CAR expression on ^U15^MCAR-NKT cells. (**E**) FACS analyses of HLA-I/II expression on ^U15^MCAR-NKT cells pre- and post-purification. (**F**) Quantification of HLA-I/II double-negative proportion of ^U15^MCAR-NKT cells (*n* = 5). (**G**) Quantification of CAR^+^ proportion of ^U15^MCAR-NKT cells (*n* = 5). (**H**) Yield of ^U15^MCAR-NKT cells (*n* = 5). (**I**) Quantification of HLA-I and HLA-II expression of ^U15^MCAR-NKT cells (*n* = 4). ^Allo15^MCAR-NKT and conventional MCAR-T cells were included as controls. (**J**-**K**) Studying the T cell-mediated allorejection of ^U15^MCAR-NKT cells. (**J**) Experimental design. (**K**) ELISA analyses of IFN-γ production on day 4 (*n* = 4). (**L**) Illustration depicting the hypoimmunogenecity working model of ^Allo/U15^MCAR-NKT cells. (**M**-**N**) Studying the in vitro antitumor efficacy of ^U15^MCAR-NKT cells against human MM and TNBC cell lines. MCAR-T cells and non-MCAR-engineered T cells were included as therapeutic cell controls. (**M**) Experimental design. (**N**) Tumor cell killing data at 24 h (*n* = 4). (**O**-**R**) Studying the in vivo antitumor efficacy of ^U15^MCAR-NKT cells in a metastatic HCC1806-FG human xenograft NSG mouse model. (**O**) Experimental design. (**P**) BLI images measuring tumor loads in experimental mice over time. (**Q**) Quantification of (**P**) (*n* = 5). (**R**) Kaplan–Meier survival curves (*n* = 5). Representative of 3 experiments. Data are presented as the mean ± SEM. ns, not significant, *p < 0.05, **p < 0.01, ***p < 0.001, ****p < 0.0001 by one-way ANOVA (I, K, and Q), two-way ANOVA (N), or log rank (Mantel-Cox) test adjusted for multiple comparisons (R)

In the first approach, co-culture of ^Allo15^MCAR-NKT cells with dissociated primary metastatic TNBC tumor samples demonstrated selective depletion of TAMs and MDSCs, attributed to their high CD1d expression (Fig. 4A–4C). Importantly, ^Allo15^MCAR-NKT cells spared other immune cell populations, including T cells, B cells, and NK cells, which expressed little to no CD1d, highlighting the specificity and safety of this approach (Fig. 4B).

In the second model, M2-polarized macrophages and MDSCs were generated from healthy donor monocytes and confirmed to express high levels of CD1d (Fig. 4D–4F and S7A-S7C). In co-culture assays, ^Allo15^MCAR-NKT cells efficiently eliminated both cell types (Fig. 4G and H, S7D, and S7E). This cytotoxicity was enhanced in the presence of α-GalCer, an NKT cell agonist, and was significantly reduced when CD1d was blocked by a neutralizing antibody, confirming a CD1d-dependent, NKT TCR-mediated killing mechanism (Fig. 4H and S7E). Notably, even in the absence of αGC, ^Allo15^MCAR-NKT cells retained cytotoxic function, suggesting an additional contribution from NKR-mediated recognition (Fig. 7H and S7E). Consistently, these cells did not harm CD1d^−^ healthy donor-derived T or B cells, further demonstrating their favorable safety profile (Fig. 4H).

In the third model, 3D TNBC tumor organoids were generated by co-culturing TNBC tumor cells with M2 macrophages or MDSCs, better mimicking the structural and immunological complexity of the TME (Fig. 4I and S7F) [43, 85]. Within these organoids, conventional MCAR-T cells exhibited limited antitumor activity, which was correlated with reduced expression of activation markers such as CD25 and diminished production of effector cytokines, including IFN-γ, TNF-α, and IL-2, indicating the functional suppression of MCAR-T cells by the immunosuppressive myeloid cells (Fig. 4J–4L, and S7G-S7J). In contrast, ^Allo15^MCAR-NKT cells maintained robust tumor cell killing and high activation status despite the presence of suppressive M2 macrophages and MDSCs (Fig. 4J–4L, and S7G-S7J). This may be due to their ability to selectively deplete these immunosuppressive cells, thereby remodeling the TME to support an effective immune response (Fig. 4K and M).

To evaluate whether NKT TCR-mediated cytotoxicity against M2-polarized macrophages and MDSCs is dependent on CAR engagement, we co-cultured these myeloid cell populations with three allogeneic NKT cell products mentioned above: ^Allo15^NKT, ^Allo15^CAR19-NKT, and ^Allo15^MCAR-NKT cells (Fig. S7K). Despite the absence of a CAR or the expression of a CD19-targeting CAR, all three NKT cell products exhibited comparable cytotoxicity against both macrophages and MDSCs (Fig. S7L). These results indicate that NKT TCR-mediated killing of CD1d^+^ myeloid cells by allogeneic NKT cells occurs independently of CAR engagement.

In summary, ^Allo15^MCAR-NKT cells exhibit a unique capacity to simultaneously target both TNBC tumor cells and key immunosuppressive components of the TME through a multifaceted CAR/TCR/NKR-mediated recognition mechanism (Fig. 4M). This tri-modal targeting strategy enables not only direct cytotoxicity toward tumor cells but also TME reprogramming, positioning ^Allo15^MCAR-NKT cells as a highly promising off-the-shelf immunotherapeutic candidate for the treatment of TNBC.

### Allogeneic MCAR-NKT cells demonstrate superior antitumor efficacy and safety in a human TNBC orthotopic xenograft model

To assess the in vivo efficacy and safety of ^Allo15^MCAR-NKT cells, we utilized a human TNBC orthotopic xenograft model in which tumor cells were implanted into the mammary fat pad of NSG mice (Fig. 5A). This model closely recapitulates the tumor growth dynamics and local tissue microenvironment of TNBC in its native anatomical context (Fig. 5A).

Both ^Allo15^MCAR-NKT cells and conventional MCAR-T cells were administered directly into the tumor site. While both therapies demonstrated significant tumor suppression, as evidenced by reduced tumor size and extended overall survival, only ^Allo15^MCAR-NKT cells achieved complete tumor elimination in all treated mice, resulting in 100% long-term survival (Fig. 5B–5E). These findings underscore the superior antitumor efficacy of ^Allo15^MCAR-NKT cells.

Notably, despite initial tumor control, several mice treated with conventional MCAR-T cells succumbed to GvHD, suggesting safety concerns associated with this approach (Fig. 5E). Flow cytometry of peripheral blood revealed that ^Allo15^MCAR-NKT cells were detectable for 2–3 weeks post-injection but cleared from circulation following tumor eradication (Fig. 5F and G). In contrast, conventional MCAR-T cells rapidly expanded and occupied ~ 50% of peripheral blood leukocytes, correlating with elevated clinical GvHD scores and histological evidence of tissue damage (Fig. 5F–5I). This expansion was associated with morbidity and mortality due to GvHD and CAR-T cell-associated toxicity (Fig. 5H and I). The seemingly shorter persistence of ^Allo15^MCAR-NKT cells may be due to their preferential localization to tumor sites with limited circulation in peripheral blood, as well as their inability to trigger GvHD-related expansion because their TCR recognizes the non-polymorphic MHC-like molecule CD1d [70, 71]. Collectively, these results demonstrate that ^Allo15^MCAR-NKT cells combine potent antitumor activity with a favorable safety profile, offering a promising therapeutic strategy for treating TNBC.

To investigate the in vivo antitumor mechanisms of ^Allo15^MCAR-NKT cells, we employed the same orthotopic TNBC model but with an increased tumor burden and compared the therapeutic efficacy of three allogeneic NKT cell products: ^Allo15^MCAR-NKT, ^Allo15^NKT, and ^Allo15^CAR19-NKT cells (Fig. S8A). Among these, only ^Allo15^MCAR-NKT cells achieved complete tumor eradication, whereas both ^Allo15^NKT and ^Allo15^CAR19-NKT cells significantly delayed tumor progression but were less effective overall (Fig. S8B and S8C). These findings suggest that while ^Allo15^NKT and ^Allo15^CAR19-NKT cells rely primarily on NKR-mediated cytotoxicity, the superior efficacy of ^Allo15^MCAR-NKT cells is likely due to the combined action of CAR-dependent and NKR-mediated tumor killing.

To further evaluate the antigen-independent antitumor activity of ^Allo15^MCAR-NKT cells, we utilized an orthotopic xenograft model with MDA-MB-231-FG human TNBC cells, which exhibit low or absent MSLN expression (Fig. S8D). This model better represents the heterogeneity and CAR antigen-low context of TNBC. In this setting, mice bearing orthotopic MDA-MB-231-FG tumors were treated with either conventional MCAR-T cells or ^Allo15^MCAR-NKT cells (Fig. S8D). As anticipated, MCAR-T cells failed to control tumor growth due to the absence of their target antigen (Fig. S8E and S8F). In contrast, ^Allo15^MCAR-NKT cells exhibited potent antitumor effects despite the lack of CAR antigen engagement, indicating their ability to eliminate tumors through antigen-independent mechanisms, likely mediated by innate-like NKRs (Fig. S8E and S8F). These results underscore the multifaceted cytotoxic potential of ^Allo15^MCAR-NKT cells and their capacity to overcome tumor antigen escape.

### Allogeneic MCAR-NKT cells demonstrate superior antitumor efficacy with strong effector function and low exhaustion in a human metastatic TNBC xenograft model

TNBC is notorious for its aggressive clinical behavior and propensity to metastasize rapidly to visceral organs, most commonly the lungs, liver, bone marrow, and brain, typically within 3–5 years of diagnosis [83]. Unlike other breast cancer subtypes, TNBC lacks actionable molecular targets and presents an immunosuppressive TME, contributing to poor therapeutic responses and high mortality [83]. To evaluate the therapeutic potential of ^Allo15^MCAR-NKT cells in metastatic TNBC, we employed a HCC1806-FG human metastatic TNBC xenograft model (Fig. 6A).

In this model, tumor cells were intravenously injected into NSG mice, initially seeding in the lungs and subsequently disseminating to distant sites, particularly the bone marrow (Fig. 6B–6G, and S9A). This recapitulates the aggressive metastatic progression observed in TNBC patients [86]. Both ^Allo15^MCAR-NKT cells and conventional MCAR-T cells were administered intravenously. While both treatments suppressed tumor progression to some extent, ^Allo15^MCAR-NKT cells achieved markedly superior tumor control and significantly prolonged survival (Fig. 6B–6G). Strikingly, bone marrow tumor burden was comparably reduced by both cell therapies; however, MCAR-T cells failed to effectively control lung-resident tumor cells. In contrast, ^Allo15^MCAR-NKT cells demonstrated robust tumor suppression in both the lungs and bone marrow, indicating broad and consistent antitumor efficacy (Fig. 6E–6G). Despite the potent initial antitumor response, tumor relapse was observed approximately 30 days following ^Allo15^MCAR-NKT cell administration, ultimately leading to animal mortality (Fig. 6B–6D). This relapse may be attributed to the limited in vivo persistence of ^Allo15^MCAR-NKT cells beyond 30 days in this model. However, given the off-the-shelf nature of this cell product, repeated dosing is feasible and may represent a viable strategy to overcome relapse and achieve sustained tumor control.

Tissue biodistribution analysis revealed that conventional MCAR-T cells predominantly localized to peripheral lymphoid organs, including the spleen and blood, with limited infiltration into the lungs, which are the primary site of TNBC tumor implantation in this model (Fig. 6H and I, and S9B). In contrast, ^Allo15^MCAR-NKT cells demonstrated preferential homing to tumor-bearing tissues, particularly the lungs, with reduced accumulation in lymphoid compartments such as the spleen and blood (Fig. 6H and I, and S9B). Additionally, a higher number of ^Allo15^MCAR-NKT cells were detected in the bone marrow, potentially due to the dissemination of metastatic HCC1806-FG tumor cells into this compartment (Fig. 6E–6I). These findings highlight the superior tumor-homing capacity of ^Allo15^MCAR-NKT cells, a critical advantage for targeting solid and metastatic tumors.

Phenotypic characterization of tumor-infiltrating therapeutic cells further supported this advantage. ^Allo15^MCAR-NKT cells displayed a highly activated and cytotoxic profile, marked by elevated expression of activation markers (i.e., CD25 and CD69), effector cytokines (i.e., IFN-γ), and cytolytic molecules (i.e., Granzyme B and Perforin) (Fig. 6J and K). Importantly, these cells maintained low expression of exhaustion markers (i.e., PD-1, CTLA-4, TIM-3, LAG-3, and TIGIT), suggesting sustained functional capacity within the immunosuppressive TME (Fig. 6L).

Collectively, these results demonstrate that ^Allo15^MCAR-NKT cells offer potent, durable antitumor efficacy in a challenging metastatic TNBC setting by combining superior tumor-homing, cytotoxicity, and resistance to functional exhaustion. These attributes position ^Allo15^MCAR-NKT cells as a promising therapeutic platform for treating metastatic TNBC.

### HLA-I/II-ablated universal MCAR-NKT cells resist host T cell-mediated allorejection while preserving potent antitumor efficacy

In addition to the risk of GvHD, a major limitation of allogeneic cell therapy is host cell-mediated allorejection. Specifically, host CD8⁺ and CD4⁺ T cells can recognize HLA class I and class II molecules, respectively, on allogeneic cells, leading to their depletion and rejection [23, 64]. This immune response can result in the premature elimination of therapeutic cells and a significant reduction in overall efficacy. To overcome this barrier, we further engineered the ^Allo15^MCAR-NKT cell product to completely eliminate surface expression of both HLA class I and class II molecules, thereby rendering the cells resistant to recognition by host CD8⁺ and CD4⁺ T cells, respectively (Fig. 7A).

This was accomplished through targeted disruption of two key genes: *B2M*, which encodes β2-microglobulin and is essential for the surface expression of all HLA class I molecules, and *CIITA*, the master transcriptional regulator of HLA class II gene expression [33, 38, 87]. Using CRISPR-Cas9-mediated gene editing with guide RNAs (gRNAs) targeting B2M and CIITA, we generated IL-15-enhanced, MCAR-NKT cells lacking both HLA-I and HLA-II expression, termed ^U15^MCAR-NKT cells (Fig. 7A).

To generate these universal CAR-NKT cells, CB-derived CD34⁺ HSPCs were first transduced with the Lenti/iNKT-MCAR-IL-15 vector (Fig. 7A and B). Simultaneously, the HSPCs were electroporated with CRISPR-Cas9 ribonucleoprotein complexes containing *B2M* and *CIITA* gRNAs. This dual genetic modification strategy consistently achieved > 50% transduction efficiency and > 50% HLA-I/II double-knockout rate (Fig. 7A and B). The engineered HSPCs were then cultured in the 6-week Ex Vivo HSPC-Derived CAR-NKT Cell Culture to generate ^U15^MCAR-NKT cells (Fig. 7C).

Importantly, ^U15^MCAR-NKT cells retained similar phenotypic characteristics, CAR expression levels, developmental trajectories, and cytotoxic profiles compared to their parental ^Allo15^MCAR-NKT counterparts (Fig. 7C–7H). The absence of HLA-I/II did not impair their differentiation or functional maturation. Furthermore, the double HLA knockout population could be further enriched through magnetic-activated cell sorting (MACS) or fluorescence-activated cell sorting (FACS) (Fig. 7E). Interestingly, even unedited ^Allo15^MCAR-NKT cells inherently expressed low levels of HLA-I and negligible HLA-II molecules, likely due to their epigenetically and transcriptionally regulated hypoimmunogenic nature within our culture system (Fig. 7E and I) [24, 32]. This feature suggests that ^Allo15^MCAR-NKT cells may already be intrinsically less immunogenic than conventional allogeneic CAR-T cells.

To evaluate immunogenicity, we performed in vitro mixed lymphocyte reaction (MLR) assays in which therapeutic cells were co-cultured with allogeneic donor PBMCs (Fig. 7J). Conventional MCAR-T cells elicited strong alloreactive T cell responses (Fig. 7K). In contrast, ^Allo15^MCAR-NKT cells induced significantly reduced T cell activation, while ^U15^MCAR-NKT cells triggered minimal to no detectable alloresponse (Fig. 7K). These data confirm that HLA-ablated ^U15^MCAR-NKT cells possess extremely low immunogenicity and are highly resistant to T cell-mediated rejection (Fig. 7L).

Importantly, despite the complete absence of HLA-I/II, ^U15^MCAR-NKT cells maintained robust antitumor activity. In vitro cytotoxicity assays demonstrated potent killing of TNBC tumor cells, and in vivo efficacy was validated in a metastatic HCC1806-FG TNBC xenograft mouse model (Fig. 7M–7R). ^U15^MCAR-NKT cells effectively suppressed tumor progression and extended survival, with comparable efficacy to ^Allo15^MCAR-NKT cells and superior performance over conventional MCAR-T cells (Fig. 6A–6D, and Fig. 7O–7R).

In summary, we have successfully developed ^U15^MCAR-NKT cells with complete ablation of HLA class I and II expression, which retain potent antitumor activity while demonstrating resistance to host T cell–mediated allorejection. In our current experimental models, including in vitro tumor cell killing assays and in vivo NSG mouse studies, both ^Allo15^MCAR-NKT and ^U15^MCAR-NKT cells exhibited comparable antitumor efficacy (Figs. 3D, 6A–6D and 7M–7R), likely due to the lack of functional host T cells in these systems. However, under physiological conditions in human patients, host CD4⁺ and CD8⁺ T cells may recognize HLA class II and class I molecules on ^Allo15^MCAR-NKT cells, respectively, leading to their immune-mediated elimination. In contrast, ^U15^MCAR-NKT cells, which lack surface HLA expression, are expected to evade such rejection. To further assess their efficacy in the presence of host immunity, advanced preclinical models, such as NSG mice reconstituted with human T cells or fully humanized NSG models, could be employed [33, 64]. Collectively, these findings underscore the therapeutic potential of ^U15^MCAR-NKT cells as a universal, off-the-shelf immunotherapy platform for the treatment of TNBC and potentially other solid tumors.

## Discussion

TNBC remains a clinically intractable malignancy due to its molecular heterogeneity, immune-evasive TME, and lack of actionable targets. Current treatment for TNBC includes anthracycline- and taxane-based chemotherapy, often with added platinum agents, while immunotherapy with anti-PD-1 antibodies such as pembrolizumab is now standard for early-stage and PD-L1⁺ metastatic TNBC in combination with chemotherapy [88, 89]. Despite advancements in chemotherapy and immunotherapy, durable clinical responses remain rare, underscoring the need for more effective and scalable cell-based therapies.

Here, we report the development and preclinical validation of a novel off-the-shelf allogeneic CAR-NKT cell therapy platform targeting MSLN, engineered from CB-derived CD34^+^ HSPCs. These allogeneic CAR-NKT cells demonstrate potent and selective cytotoxicity against TNBC, favorable biodistribution, and an improved safety profile, positioning them as a next-generation immunotherapy for TNBC.

Our platform integrates efficient lentiviral TCR/CAR transduction, feeder-free ex vivo differentiation, and gene engineering to generate high-yield, high-purity therapeutic products across multiple CB donors [24]. The final products co-express a third-generation MSLN-specific CAR, an invariant NKT TCR, and soluble IL-15 to support expansion and persistence. Notably, our culture system does not produce CD4 SP CAR-NKT cells, but instead yields CD8 SP and DN subsets. This phenomenon is commonly observed in many ex vivo stem cell–derived T cell and NKT cell differentiation systems [38, 90–92]. During differentiation, the transition from DP to CD4 or CD8 SP stages—representing a critical step of functional maturation known as positive selection—is not yet fully recapitulated in vitro. Most feeder-free protocols rely on anti-CD3 antibody stimulation, which delivers supraphysiological TCR signals and drives agonist selection, often resulting in innate-like T cell phenotypes [93, 94]. Furthermore, current stem cell differentiation systems employ Notch signaling to induce T lineage specification. However, Notch activation skews the CD4/CD8 lineage decision, preferentially promoting CD8⁺ T cell development over CD4⁺ T cells [95, 96].

Besides CB HSPCs, peripheral blood–mobilized CD34⁺ HSPCs can also serve as a source to generate allogeneic CAR-NKT cells, offering the advantage of higher HSPC yield and easier accessibility from healthy adult donors, which facilitates large-scale manufacturing and donor screening for optimal HLA matching or immune compatibility [38, 97, 98]. Although the current study did not evaluate peripheral blood–mobilized CD34⁺ HSPCs, future investigations directly comparing CAR-NKT cell products derived from different HSPC sources would be of significant interest.

During the differentiation of allogeneic MCAR-NKT cells, we employed a feeder-free system for early-stage development, supporting translational and clinical scalability. In the final maturation step, a K562-based feeder cell expansion method was utilized. We acknowledge that the use of feeder cells carries certain potential risks, including the possibility of genomic material transfer, variability in cell product consistency, and challenges in regulatory compliance for large-scale manufacturing [99]. However, K562-based feeder cell systems have been widely adopted in clinical trials, particularly for the expansion of human CAR-NK cell products, and have demonstrated both clinical feasibility and safety [71, 100–103]. Alternative feeder-free expansion strategies, such as αCD3/αCD28-based stimulation, could also be applied to further enhance clinical compatibility [40, 104]. Importantly, in our protocol, allogeneic MCAR-NKT cells were stimulated only once with K562 feeder cells, as repeated stimulation with aAPCs may be associated with increased risk of CAR-NKT cell exhaustion. Using this optimized feeder-free differentiation and feeder-dependent expansion approach, we successfully generated ^Allo15^MCAR-NKT cells with high yield, purity, and functional potency, which is suitable for therapeutic application in patients with TNBC.

Using primary TNBC patient samples, we performed immune profiling of TNBC tumor and TME compartments (Fig. 1). Tumor cells exhibited high but heterogeneous expression of known CAR targets including MSLN, TROP2, EGFR, and Nectin-4, with MSLN and TROP2 emerging as promising candidates due to their high expression on tumor cells and minimal presence in normal tissues. Our study focused on MSLN-specific ^Allo15^MCAR-NKT cells as a proof-of-concept, given MSLN’s clinical relevance across multiple solid tumors such as ovarian, pancreatic, and lung cancers [105–107]. The success of this approach establishes a foundation for expanding the platform to target other solid tumor types. Importantly, the robustness and versatility of our HSPC-derived CAR-NKT cell platform make it well-suited for the development of additional allogeneic CAR-NKT cell products, such as TROP2-targeting ones, in future studies. On the other hand, TNBC tumor cells also expressed ligands for activating NKRs (e.g., MICA/B, ULBPs, CD112, and CD155), suggesting a role for innate-like NKR-mediated killing mechanisms. Compared to conventional CAR-T cells, ^Allo15^MCAR-NKT cells exhibited superior cytotoxicity against TNBC cells in vitro, driven by both CAR- and NKR-mediated mechanisms (Fig. 3). In this manner, ^Allo15^MCAR-NKT cells overcame immune evasion through downregulation of MSLN, a key mode of resistance to CAR-T cell therapy, by co-engaging innate NKRs to target antigen-low or antigen-negative tumor populations [78].

The TNBC TME is densely infiltrated with immunosuppressive myeloid cells, particularly TAMs and MDSCs, which play a central role in mediating immune evasion [108, 109]. These cells suppress the function of cytotoxic T cells and natural killer cells through multiple mechanisms, including secretion of inhibitory cytokines (e.g., IL-10 and TGF-β), upregulation of immune checkpoint ligands, and metabolic reprogramming that depletes nutrients essential for effector immune cells [110]. As a result, the presence of TAMs and MDSCs fosters immune exclusion and promotes resistance to both chemotherapy and conventional immunotherapies, such as checkpoint blockade [111]. Therefore, overcoming this immunosuppressive barrier is critical for the success of next-generation immunotherapeutic strategies in TNBC. Our study reveal that ^Allo15^MCAR-NKT cells uniquely target these CD1d^+^ immune suppressive cells via their invariant NKT TCR. In vitro co-culture experiments demonstrated selective elimination of TAMs and MDSCs, which was abrogated by CD1d blockade (Fig. 4). This TME remodeling capacity was not observed with conventional CAR-T cells, indicating that NKT-specific interactions play a critical role in modulating the TNBC TME. These findings support the notion that allogeneic CAR-NKT cells can reshape the TME by directly depleting immunosuppressive populations, thereby enhancing overall antitumor efficacy.

The route of administration is an important consideration in the design of immune-cell therapies for TNBC. Intravenous infusion is the most common approach for its systemic reach and scalability, particularly for patients with metastatic disease. However, it may result in limited tumor infiltration, off-target accumulation, and systemic toxicities [112]. In contrast, locoregional delivery—such as intratumoral or peritumoral injection—offers enhanced tumor exposure with reduced systemic distribution. It is suited for early-stage, localized breast cancers, where direct access to the tumor site is feasible (NCT00861107, NCT01837602, NCT02061332) [113–115]. Due to the heterogeneity of breast cancer progression, a combination administration strategy, such as local priming followed by systemic administration, could further potentiate therapeutic outcomes by balancing local cytotoxicity with systemic immune surveillance. In our preclinical models, we simulated localized disease using an orthotopic mammary fat pad TNBC model followed by peritumoral injection of therapeutic cells (Fig. 5). In this setting, ^Allo15^MCAR-NKT cells demonstrated robust antitumor activity with minimal GvHD risk. To model metastatic cancer, we established a breast cancer metastasis model by intravenously injecting breast cancer cells, followed by systemic administration of ^Allo15^MCAR-NKT cells (Fig. 6). In this model, ^Allo15^MCAR-NKT cells effectively homed to major metastatic sites, including the lung, liver, and bone marrow, and mediated tumor control.

One of the major obstacles of adoptive cell therapy is the safe and effective development of allogeneic cell products [21]. Efforts to develop allogeneic CAR-T therapies have been met with substantial hurdles. Conventional αβ T cells from third-party donors pose a risk of GvHD, unless their endogenous TCRs are genetically disrupted. While gene editing strategies (e.g., *TRAC* knockout) have shown promise, they add complexity, increase manufacturing costs, and raise safety concerns related to genomic instability and off-target effects [116]. Several platforms have been developed to address this challenge. TALEN-edited universal CAR-T cells (e.g., ALLO-501) reduce GvHD and allorejection by eliminating TCR and HLA expression, but rely on extensive genome editing, increasing manufacturing complexity [117–120]. iPSC-derived CAR-NK and CAR-T therapies offer scalable sources of allogeneic effectors with low GvHD risk, yet face challenges related to limited persistence, cytokine dependence, and differentiation variability [100, 102, 121–124].

NKT cells recognize lipid antigens presented by the non-polymorphic CD1d molecule, rather than conventional peptide-HLA complexes. Importantly, human NKT cells do not cause GvHD due to their restricted TCR usage, limited proliferative response against allogeneic tissues, and low alloreactive potential [65, 125–127]. Their invariant nature makes them an ideal scaffold for building allogeneic CAR-based therapies without the need for TCR knockout. In our study, ^Allo15^MCAR-NKT cells localized within the tumor region and showed minimal entry into the peripheral circulation, likely due to their tissue-resident phenotype and chemokine receptor expression profile (Fig. 5) [32]. This tumor-specific biodistribution may reduce the risk of systemic toxicity, a major challenge in CAR-T cell therapy [128, 129]. In addition, ^Allo15^MCAR-NKT cells demonstrated minimal signs of GvHD, as evidenced by low clinical scores, limited expansion in blood, and negligible elevation of liver or kidney damage markers (Figs. 5 and 6). In contrast, conventional MCAR-T cells accumulated in the periphery over time and were associated with elevated GvHD markers, consistent with known risks of allogeneic CAR-T therapy (Figs. 5 and 6) [21, 22]. Notably, we acknowledge that the GvHD-associated toxicities observed in MCAR-T cell–treated mice within the xenograft model results from xenogeneic GvHD, which is not relevant to patients receiving autologous CAR-T cell therapy. However, this risk becomes clinically significant in the context of allogeneic CAR-T cell therapies [63]. In our study, MCAR-T cells were primarily used as a benchmark to underscore the GvHD-free profile and enhanced safety of ^Allo15^MCAR-NKT cells, supporting their promise as a safe and effective allogeneic cell therapy for the treatment of TNBC.

While the development of off-the-shelf allogeneic cell therapies offers major logistical and clinical advantages over autologous approaches, a persistent challenge is the host cell-medaited allorejection [21, 22]. In allogeneic T cell-based therapies, allorejection is primarily driven by host CD8⁺ and CD4⁺ T cells, which recognize foreign HLA class I and II molecules, respectively, on the surface of therapeutic cells [21, 22]. This immune clearance dramatically reduces cell persistence and therapeutic efficacy, especially in solid tumors, where repeated or prolonged engagement is required for adequate antitumor responses. Therefore, we engineered a universal variant of the ^Allo15^MCAR-NKT cell product (^U15^MCAR-NKT) by knocking out HLA-I and HLA-II via CRISPR-Cas9 targeting of *B2M* and *CIITA* loci in HSPCs prior to differentiation (Fig. 7). This strategy yielded pure, potent CAR-NKT cell products lacking alloimmune-activating HLA molecules. ^U15^MCAR-NKT cells retained the antitumor activity in vitro and in vivo, while significantly reducing allogeneic T cell responses in the MLR assays. These data support the feasibility of universal, off-the-shelf CAR-NKT immunotherapy with minimized risk of immune rejection or host-vs-graft responses.

While our study provides compelling evidence for the efficacy and safety of ^Allo15^MCAR-NKT cells in preclinical TNBC models, several limitations remain. First, although we modeled both orthotopic and metastatic TNBC, patient-derived xenografts (PDXs) with greater intratumoral heterogeneity and immune complexity may be needed to further validate our findings [130]. Second, long-term persistence and memory potential of ^Allo15^MCAR-NKT cells require further investigation, particularly in the context of potential tumor recurrence or immune reconstitution post-treatment. third, with this versatile platform, our future studies will explore multiplex engineering approaches incorporating immunostimulatory payloads (e.g., IL-12, IL-18, and IL-21), metabolic modulators (e.g., PGC-1α and GOT2), or additional CAR constructs targeting secondary antigens (e.g., CARs targeting TROP2 and EGFR) to enhance persistence, cytotoxicity, and resistance to exhaustion within the hostile TNBC TME [131–135]. In addition, iPSCs offer an unlimited source and are highly amenable to multiplex gene editing, making them an attractive platform for generating off-the-shelf CAR-NKT cells. This approach could facilitate scalable manufacturing and enhance the potential for pharmaceutical development and industrialization. Finally, in our study, we utilized a third-generation MCAR construct incorporating CD28–4-1BB–CD3ζ intracellular signaling domains, originally optimized for αβ T cells. However, it remains unclear whether this configuration is optimal for NKT cell biology. It will be important in future studies to explore alternative CAR designs incorporating signaling domains more tailored to NKT cells, such as 2B4, CD27, or ICOS, which may better support their activation, persistence, and functionality [101, 136, 137]. Moreover, the potential synergy between CAR, invariant TCR, and NKR signaling pathways presents an intriguing area for further investigation, as concurrent engagement of these pathways may induce unique transcriptional or metabolic programs that enhance therapeutic potency of ^Allo15^MCAR-NKT cells.

## Conclusions

In this study, we developed a clinically translatable allogeneic CAR-NKT cell therapy platform by engineering human CD34⁺ HSPCs with a mesothelin-specific CAR and NKT TCR, followed by a feeder-free culture system to generate ^Allo15^MCAR-NKT cells. These cells were produced at high yield, purity, and consistency, offering a scalable and robust “off-the-shelf” therapeutic product. Compared to conventional MCAR-T cells, ^Allo15^MCAR-NKT cells demonstrated superior antitumor efficacy across multiple in vitro and in vivo TNBC models. This enhanced activity is supported by multiple tumor-killing mechanisms (CAR-, NKR-, and TCR-mediated), improved tumor-homing, elevated effector functions, and reduced exhaustion. Importantly, they exhibited a favorable safety profile with no signs of GvHD. To further improve persistence and reduce immunogenicity, we generated ^U15^MCAR-NKT cells through CRISPR-mediated HLA-I/II ablation. These universal cells resisted host T cell-mediated allorejection while maintaining potent antitumor effects. Altogether, our results establish ^Allo/U15^MCAR-NKT cells as a potent and safe immunotherapy with broad applicability for TNBC treatment, particularly in addressing tumor heterogeneity and immune suppression. This platform holds strong promise for clinical translation as a next-generation, off-the-shelf cell therapy for solid tumors.

## Supplementary Information

Below is the link to the electronic supplementary material.

Supplementary Material new

## Data Availability

The data that support the findings of this study are available on request from the corresponding author. The data are not publicly available due to privacy or ethical restrictions. The scRNA-seq data generated or reanalyzed in this study have been deposited in the public repository Gene Expression Omnibus Database (GSE304213 and GSE241996).

## Abbreviations

ADC: Antibody-drug conjugate
^Allo15^CAR19-NKT: Allogeneic IL-15-enhanced CD19-targeting CAR-engineered NKT
^Allo15^MCAR-NKT: Allogeneic IL-15-enhanced MCAR-engineered NKT
^Allo15^NKT: Allogeneic IL-15-enhanced NKT
^Allo^MCAR-NKT: Allogeneic MCAR-engineered NKT
BLI: Bioluminescence live animal imaging
CAR: Chimeric antigen receptor
CAR-T: CAR-engineered T
CB: Cord blood
DN: Double-negative
DP: Double-positive
EGFR: Epidermal growth factor receptor
ER: Estrogen receptor
FACS: Fluorescence-activated cell sorting
GvHD: Graft-versus-host disease
HSPC: Hematopoietic stem and progenitor cell
ICI: Immune checkpoint inhibitor
MACS: Magnetic-activated cell sorting
MCAR: MSLN-targeting CAR
MCAR-T: MCAR-engineered T
MDSC: Myeloid-derived suppressor cell
MLR: Mixed lymphocyte reaction
MM: Multiple myeloma
MSLN: Mesothelin
NKR: Natural killer receptor
NKT: Natural killer T
OS: Overall survival
PDXs: Patient-derived xenografts
PR: Progesterone receptor
scRNA-seq: Single-cell RNA sequencing
SP: Single-positive
TAM: Tumor-associated macrophage
TME: Tumor microenvironment
TNBC: Triple-negative breast cancer
^U15^MCAR-NKT: Universal IL-15-enhanced MCAR-engineered NKT
VCN: Vector copy number
