# Supplementary material for: Targeting triple-negative breast cancer using cord-blood CD34⁺ HSPC-derived mesothelin-specific CAR-NKT cells with potent antitumor activity
Source: J Hematol Oncol. 2025 Oct 13;18:86. doi: 10.1186/s13045-025-01736-9 (PMC12516852; doi:10.1186/s13045-025-01736-9)
Supplement: Supplementary file 1 — Supplementary Material new [file 13045_2025_1736_MOESM1_ESM.docx]

**Supplementary Information**

**Targeting triple-negative breast cancer using cord-blood CD34⁺ HSPC-derived mesothelin-specific CAR-NKT cells with potent antitumor activity**

Yan-Ruide Li^1,2^, Xinyuan Shen^1,2^, Yichen Zhu^1,2^, Zhe Li^1,2^, Ryan Hon^1,2^, Yanxin Tian^1,2^, Jie Huang^1,2^, Annabel S. Zhao^1,2^, Nathan Y. Ma^1,2^, Catherine Zhang^1,2^, David Lin^3^, Karine Sargsyan^4^, Yuan Yuan^3^, Lili Yang^1,2,5,6,7,8,9,§^

**Author Affiliation:**

^1^Department of Microbiology, Immunology & Molecular Genetics, University of California, Los Angeles, Los Angeles, CA 90095, USA

^2^Department of Bioengineering, University of California, Los Angeles, Los Angeles, CA 90095, USA

^3^Division of Medical Oncology, Cedars-Sinai Medical Center, Los Angeles, CA 90048, USA

^4^OncoBiobank, Cedars-Sinai Medical Center, Los Angeles, CA 90048, USA

^5^Eli and Edythe Broad Center of Regenerative Medicine and Stem Cell Research, University of California, Los Angeles, Los Angeles, CA 90095, USA

^6^Jonsson Comprehensive Cancer Center, David Geffen School of Medicine, University of California, Los Angeles, Los Angeles, CA 90095, USA

^7^Molecular Biology Institute, University of California, Los Angeles, CA 90095, USA

^8^Parker Institute for Cancer Immunotherapy, University of California, Los Angeles, Los Angeles, CA 90095, USA

^9^Goodman-Luskin Microbiome Center, University of California, Los Angeles, Los Angeles, CA 90095, USA

^§^Corresponding author. Email: [liliyang@ucla.edu](mailto:liliyang@ucla.edu) (L.Y.)

**

**

**Fig.S1. Immune profiling of TNBC tumor cells and immune cells in the TME; related to Fig. 1.**

(A) FACS gating strategy of TNBC tumor cells and immune cells. Mono, monocyte; TAM, tumor-associated macrophage; MDSC, myeloid-derived suppressor cell; NK, natural killer cell; FAP, fibroblast activation protein.

(B) FACS analyses of EpCAM expression on tumor cells from TNBC patient samples. EpCAM, epithelial cell adhesion molecule.

(C) FACS detection of NKT cells within the total CD3^+^ T cells.





**Fig. S2. Generation and characterization of ^Allo15^MCAR-NKT cells and PBMC-derived MCAR-T cells; related to Fig. 2.**

(A) FACS detection of NKT TCR expression in CD34^+^ HSPCs from all seven cord blood donors at 72 h after lentivector transduction. Intracellular staining with anti-TCR Vβ11 antibody was used to detect NKT TCR expression.

(B-D) Studying the functional contribution of IL-15 to ^Allo15^MCAR-NKT cells. (B) ELISA analyses of IL-15 production by ^Allo15^MCAR-NKT cells (n = 4). ^Allo15^MCAR-NKT cells were cultured *in vitro* for 24 hours with αGC-pulsed PBMCs in the absence of exogenous cytokine supplementation, and the supernatant were collected for ELISA analysis. (C) Experimental design to study ^Allo15^MCAR-NKT cell proliferation with or without exogenous cytokine supplementation. (D) Quantification of live ^Allo15^MCAR-NKT cells over time (n = 4).

(E-I) Generating conventional MCAR-T cells. (E) Schematics showing the design of Lenti/MCAR vector and the generation of MCAR-T cells. (F) FACS analyses of CAR and CD4/CD8 coreceptor expression on MCAR-T cells. (G) Yield of MCAR-T cells (n = 7). (H) Quantification of CAR^+^ proportion of MCAR-T cells (n = 7). (I) Quantification of MSLN^+^ proportion of the indicated cells (MCAR-T and ^Allo15^MCAR-NKT cells, n = 7; T and ^Allo15^NKT cells, n = 5).

(J-K) Studying the KIR expression on ^Allo15^MCAR-NKT and MCAR-T cells. (J) FACS detection of KIR expression on the indicated cells. PBMC-derived NK cells were included as a control. (K) Quantification of (J) (n = 5; n indicates different CB or PBMC donors).

Representative of over 5 experiments. Data are presented as the mean ± SEM. ns, not significant, ****p < 0.0001 by Student’s *t* test (B), one-way ANOVA (J), or two-way ANOVA (D).





**Fig. S3. Transcriptional profiling of ^Allo15^MCAR-NKT cells; related to Fig. 2.**

(A) Violin plots showing the expression distribution of the indicated transcription factor (TF) genes in ^Allo15^MCAR-NKT and conventional MCAR-T cells.

(B) Violin plots showing the expression distribution of the indicated TF genes in allogeneic HSPC-derived CAR-NKT cells during their development. Note that we used previously generated data on the development of HSPC-derived BCMA-targeting CAR-engineered NKT cells (Gene Expression Omnibus Database, GSE241996). No significant differences were observed among HSPC-derived CAR-NKT cells expressing different CAR constructs.

(C-F) Comparison between ^Allo15^MCAR-NKT and healthy donor PBMC-derived IL-15-engineered MCAR-NKT (^PBMC15^MCAR-NKT) cells using scRNA-seq.

(C) Schematics showing the generation and scRNA-seq analyses of ^PBMC15^MCAR-NKT cells.

(D and E) Violin plots showing the expression distribution of the indicated genes and gene signatures in ^Allo15^MCAR-NKT and ^PBMC15^MCAR-NKT cells.

(F) Pathway analyses of differentiated expressed genes comparing ^Allo15^MCAR-NKT with ^PBMC15^MCAR-NKT cells.

**

**

**Fig. S4. Evaluation of antigen-independent cytokine release by ^Allo15^MCAR-NKT cells; related to Fig. 3.**

(A) Experimental design. Healthy donor PBMC-derived B cells were selected as a target cell control because they are not killed by ^Allo15^MCAR-NKT cells and, unlike T cells, secrete minimal effector cytokines, reducing background noise.

(B) ELISA measurements of cytokine levels in culture supernatants collected 24 hours after co-culture with the indicated target cells (n = 4).

Representative of 3 experiments. Data are presented as the mean ± SEM. ns, not significant, *p < 0.05, ****p < 0.0001 by one-way ANOVA.

**

**

**Fig. S5. Generation of ^Allo15^NKT and ^Allo15^CAR19-NKT cells and evaluation of their *in vitro* antitumor capacity; related to Fig. 3.**

(A) Schematics showing the design of lentivectors and the generation of ^Allo15^NKT and ^Allo15^CAR19-NKT cells.

(B-C) Studying the *in vitro* antitumor capacity of ^Allo15^NKT, ^Allo15^CAR19-NKT, and ^Allo15^MCAR-NKT cells. (B) Experimental design. (C) Tumor cell killing data at 24 h (n = 4).

(D-E) Studying the *in vitro* tumor cell killing mechanisms of ^Allo15^NKT and ^Allo15^CAR19-NKT cells. (D) Experimental design. (E) Tumor cell killing data at 24 h (E:T ratio = 2:1; n = 4).

Representative of 3 experiments. Data are presented as the mean ± SEM. ns, not significant, ****p < 0.0001 by two-way ANOVA (C), or one-way ANOVA (E).

**

**

**Fig. S6. Evaluation of the long-term tumor cell killing capacity and antigen escape resistance of ^Allo15^MCAR-NKT cells; related to Fig. 3.**

(A) ELISA analyses of IFN-γ production in ^Allo15^MCAR-NKT and MCAR-T cells, following one-time, three-time, and five-time tumor cell co-cultures (n = 4).

(B-G) Comparison between ^Allo15^MCAR-NKT and non–IL-15-enhanced allogeneic MCAR-NKT (^Allo^MCAR-NKT) cells. (B) Schematics showing the design of lentivectors and the generation of ^Allo^MCAR-NKT cells. (C) FACS detection of the purity and MCAR expression of ^Allo^MCAR-NKT and ^Allo15^MCAR-NKT cells. (D) Quantification of CAR^+^ proportion of ^Allo^MCAR-NKT and ^Allo15^MCAR-NKT cells (n = 3). (E) Yield of ^Allo^MCAR-NKT and ^Allo15^MCAR-NKT cells (n = 3). (F and G) Studying the long-term tumor cell killing of ^Allo^MCAR-NKT and ^Allo15^MCAR-NKT cells. (F) Experimental design. Note the stimulator cells were not labeled with FG while the indicator cells were labeled with FG. (G) Tumor cell killing data (n = 4).

(H-K) Generation of a TNBC cell line model to study CAR antigen expression and escape for evaluating ^Allo15^MCAR-NKT cell efficacy. (H) Schematics showing the indicated human TNBC cell line. MDA-MB-231-MSLN-FG, MDA-MB-231-FG cell engineered to overexpress MSLN. (I) FACS analyses of MSLN expression on MDA-MB-231-MSLN-FG cells. (J) Experimental design. (K) Tumor cell killing data at 24 h (n = 4). Note that MCAR-T cells demonstrated enhanced cytotoxicity with increasing effector-to-target ratios, suggesting their potential efficacy at higher cell doses.

(L-N) Evaluation of susceptibility of MSLN^high^ and MSLN^low^ primary tumor cells to ^Allo15^MCAR-NKT cell-mediated killing. (L) Experimental design. (M) FACS detection of MSLN expression on the FACS-sorted tumor cells. (N) Tumor cell killing data at 24 h (E:T ratio = 1:1; n = 3).

Representative of 3 experiments. Data are presented as the mean ± SEM. **p < 0.01, ***p < 0.001, ****p < 0.0001 by one-way ANOVA (A and N), Student’s *t* test (D and E), or two-way ANOVA (G and K).

**

**

**Fig. S7. Studying the CD1d^+^ immunosuppressive myeloid cell targeting by ^Allo15^MCAR-NKT cells; related to Fig. 4.**

(A-C) *In vitro* generation of human MDSCs. (A) Experimental design. GM-CSF, granulocyte-macrophage colony-stimulating factor. (B) FACS detection of CD1d on MDSCs. Healthy donor PBMC-derived T and B cells were included as staining controls. (C) Quantification of (B) (n = 5).

(D-E) Studying the MDSC targeting by ^Allo15^MCAR-NKT cells. (D) Experimental design. (E) FACS analyses of live MDSCs at 24 h after co-culturing with ^Allo15^MCAR-NKT cells (n = 4). Live cells were identified as e506^−^CD14^+^CD11b^+^ cells. An anti-CD1d antibody was added into the coculture to block CD1d/NKT TCR recognition.

(F-J) Studying the MDSC targeting by ^Allo15^MCAR-NKT cells using an *in vitro* 3D tumor organoid culture. (F) Experimental design. (G) Tumor killing data at 24 h (*n* = 4). (H) MDSC killing data at 24 h (n = 4). (I) FACS analyses of surface activation marker (i.e., CD25) expression on the indicated therapeutic cells (n = 4). (J) FACS analyses of intracellular effector cytokine (i.e., IFN-γ, TNF-α, and IL-2) production by the indicated therapeutic cells (n = 4).

(K-L) Studying the M2-polarized macrophage and MDSC targeting by ^Allo15^NKT, ^Allo15^CAR19-NKT, and ^Allo15^MCAR-NKT cells. (K) Experimental design. (L) FACS analyses of live macrophages or MDSCs at 24 h after co-culturing with the indicated allogeneic NKT cells (n = 4).

Representative of 3 experiments. Data are presented as the mean ± SEM. ns, not significant, *p < 0.05, **p < 0.01, ***p < 0.001, ****p < 0.0001 by Student’s *t* test (I left), or by one-way ANOVA (C, E, G, H, I right, J, and L).

**

**

**Fig. S8. Evaluation of the *in vivo* antitumor efficacy of ^Allo15^MCAR-NKT cells in human orthotopic TNBC xenograft models.**

(A-C) Evaluation of the *in vivo* antitumor efficacy of ^Allo15^NKT, ^Allo15^CAR19-NKT, and ^Allo15^MCAR-NKT cells. (A) Experimental design. An orthotopic HCC1806-FG human xenograft NSG mouse model was utilized. (B) BLI images measuring tumor loads in experimental mice over time. (C) Quantification of (B) (n = 3).

(D-F) Evaluation of the *in vivo* antitumor efficacy of ^Allo15^MCAR-NKT and conventional MCAR-T cells. (D) Experimental design. An orthotopic MDA-MB-231-FG human xenograft NSG mouse model was utilized. (E) BLI images measuring tumor loads in experimental mice over time. (F) Quantification of (E) (n = 4).

Representative of 2 experiments. Data are presented as the mean ± SEM. ns, not significant, **p < 0.01, ***p < 0.001, ****p < 0.0001 by one-way ANOVA.

**

**

**Fig. S9. Evaluation of the *in vivo* antitumor efficacy of ^Allo15^MCAR-NKT cells in a human metastatic TNBC xenograft model.**

(A) BLI images measuring tumor loads in all experimental mice over time. Data correspond to Figure 6B and 6E. The image data from mouse #1 is presented in Figure 6B, and the data from mouse #2 is presented in Figure 6E.

(B) FACS analyses of CD56 and MCAR expression on the indicated therapeutic cells isolated from the lungs of experimental mice. Data correspond to Figure 6H. MCAR-T cells were identified as CD3⁺CD45⁺CD56⁻MCAR⁺, whereas ^Allo15^MCAR-NKT cells were CD3⁺CD45⁺CD56⁺MCAR⁺, confirming their respective phenotypic identities.

**Table S1. Primary metastatic TNBC patient information**

| Sample ID | Age | Tumor stage | ER | PR | HER2 IHC | HER2 FISH | Ki67 |
| --- | --- | --- | --- | --- | --- | --- | --- |
| 1 | 52 | Stage 4 | 0% | 0% | 0 | Negative | 69% |
| 2 | 38 | Stage 4 | <1% | <1% | 1+ | Negative | 96% |
| 3 | 37 | Stage 4 | 0% | 15% | 0 | Negative | 78% |
| 4 | 37 | Stage 4 | 0% | 0% | 2+ | Negative | 79% |
| 5 | 63 | Stage 4 | <1% | 2% | 0 | Negative | 47% |

Abbreviations: ER, estrogen receptor; PR, progesterone receptor; HER2, human epidermal growth factor receptor 2; IHC, Immunohistochemistry; FISH, fluorescence *in situ* hybridization.
